# Mechanistic Insights into Unary Peptide–Membrane Interactions Enable Stable Encapsulation and Trigger-Responsive Peptidyl Liposomes

**DOI:** 10.1021/jacs.5c21158

**Published:** 2026-02-18

**Authors:** Hua-De Gao, Jia-Lin Hong, Cheng-Bang Jian, Tzu-Ho Chen, Ning-Chu Chang, Suthasinee Meeroekyai, Ruei-Yu He, Yi-Ting Liao, Chun-Hsiung Wang, Chun-Jen Su, U-Ser Jeng, Meng-Chiao Ho, Yu-Ju Chen, Hsien-Ming Lee

**Affiliations:** † Institute of Chemistry, 38017 Academia Sinica, Taipei 11529, Taiwan; ‡ Department of Chemistry, 33561 National Taiwan University, Taipei 10617, Taiwan; § Nano Science and Technology Program, Taiwan International Graduate Program, Academia Sinica, Taipei 11529, Taiwan; ∥ Chemical Biology and Molecular Biophysics Program, Taiwan International Graduate Program, Academia Sinica, Taipei 11529, Taiwan; ⊥ Institute of Biological Chemistry, Academia Sinica, Taipei 11529, Taiwan; # 57815 National Synchrotron Radiation Research Center, Hsinchu 300092, Taiwan

## Abstract

Efforts to engineer trigger-responsive peptidyl liposomes have historically been limited by premature release following peptide conjugation, reflecting an incomplete understanding of tethered peptide–membrane interactions. Here, we establish a unified mechanistic framework for designing encapsulation-stable yet trigger-responsive liposomes by elucidating how membrane-anchored peptides interact with membranes. Screening identified MAG2 as, thus far, the only AMP backbone that can be surface-masked without liposome leakage while preserving latent lytic activity—-an outcome previously unattainable in unary peptidyl liposome systems. Cryo-EM/cryo-ET, SAXS, CD, FLIM/fluorescence imaging, and MTT assays collectively unveil a hierarchical cascade of molecular events: masked peptide–membrane conjugation with stable liposome encapsulation, lateral-diffusion-driven outer leaflet PEG-layer expansion, trigger-induced peptide unmasking, aggregation-mediated membrane defect formation, and outer leaflet PEG-layer collapse, culminating in liposomal content release, intracellular distribution, and trigger-induced cell-killing efficacy. These findings uncover a unary peptide–membrane interaction mechanism that will redefine the design principles of trigger-responsive therapeutic peptidyl liposomes.

## Graphical abstract

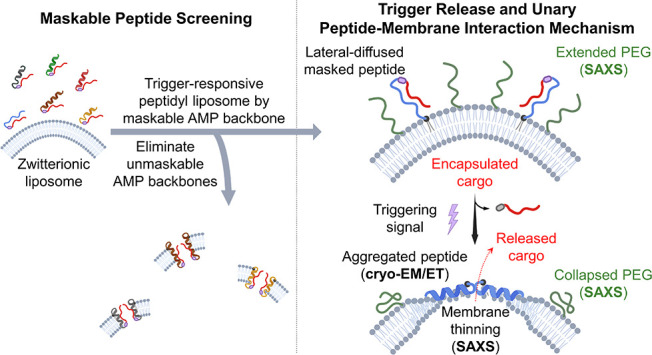

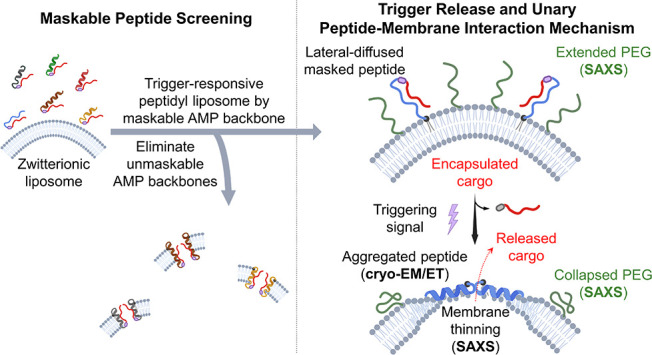

## Introduction

While stably encapsulating chemotherapeutics within liposomes can diminish their detrimental effects on healthy tissues,^1,2^ the impeded drug release significantly reduces therapeutic efficacy.^3,4^ Some technical advances for activating liposomal release at intended timing and location with precise chemical/biochemical cues,^2^ including ex vivo stimuli (light,^5–7^ temperature,^8^ ultrasound,^9,10^ and magnetic field^11,12^ ) and a few in vivo stimuli (pH,^13–15^ redox potential,^16^ and enzymes^17–20^ ), have been developed relying on less compatible complex artificial lipids.^21,22^ In nature, evolutionary pressure has favored proteins and peptides, rather than lipid structural complexity, as the primary regulators of biological processes and mediators of membrane functions. Their inherent ability to recognize and respond to diverse biochemical signals suggests that trigger-responsive membrane-lytic peptides can be engineered into liposomal systems to achieve safer, more efficient, and biocompatible drug delivery. This strategy holds promise for advancing liposome-based therapeutics.

All antimicrobial peptides (AMPs) can disrupt negatively charged bacterial membranes, while some are also capable of disrupting zwitterionic mammalian cell membranes. AMPs typically comprise cationic and hydrophobic amino acids and usually fold into an amphiphilic α-helix upon interaction with membranes.^23,24^ The well-aligned positively charged and hydrophobic amino acids form amphiphilic helix that can promote peptide lateral aggregation and disrupt membranes by forming transmembrane pores or membrane micellization,^25–27^ presenting a compelling avenue for developing liposomal trigger-responsive release. The primary challenge in trigger-responsive peptidyl liposome release lies not only in release efficacy but also in ensuring complete trigger-waiting encapsulation stability. Despite various AMP masking strategies, significant premature release after peptide–liposome conjugation (to form a unary system) has hindered the development of successful unary trigger-responsive AMP peptidyl liposomes,^5,18,28^ with the sole exception being pH-triggered systems.^29^ This challenge likely arises from the choice of AMP backbones rather than masking strategies, underscoring the need to understand peptide–membrane interactions within covalently linked, membrane-confined environments. Peptide conjugation drastically elevates the effective local concentration of peptides at the membrane due to proximity effects, making their membrane lyticity more difficult to suppress compared with classical thermodynamic peptide–membrane equilibria in binary systems. While such binary systems can maintain encapsulation stability before masking, they are impractical for trigger-responsive drug delivery. Therefore, this work aims to identify an AMP backbone that ensures complete trigger-waiting encapsulation stability when conjugated to liposomes yet rapidly disrupts membranes upon activation. Moreover, we elucidate the mechanism of AMP–membrane interaction in this conjugated, or “unary” mode. Our design integrates three essential elements—a membrane-lytic AMP domain, a trigger-responsive linker, and a masking domain that suppresses peptide lyticity prior to activation (Figure 1).

**Figure 1:**
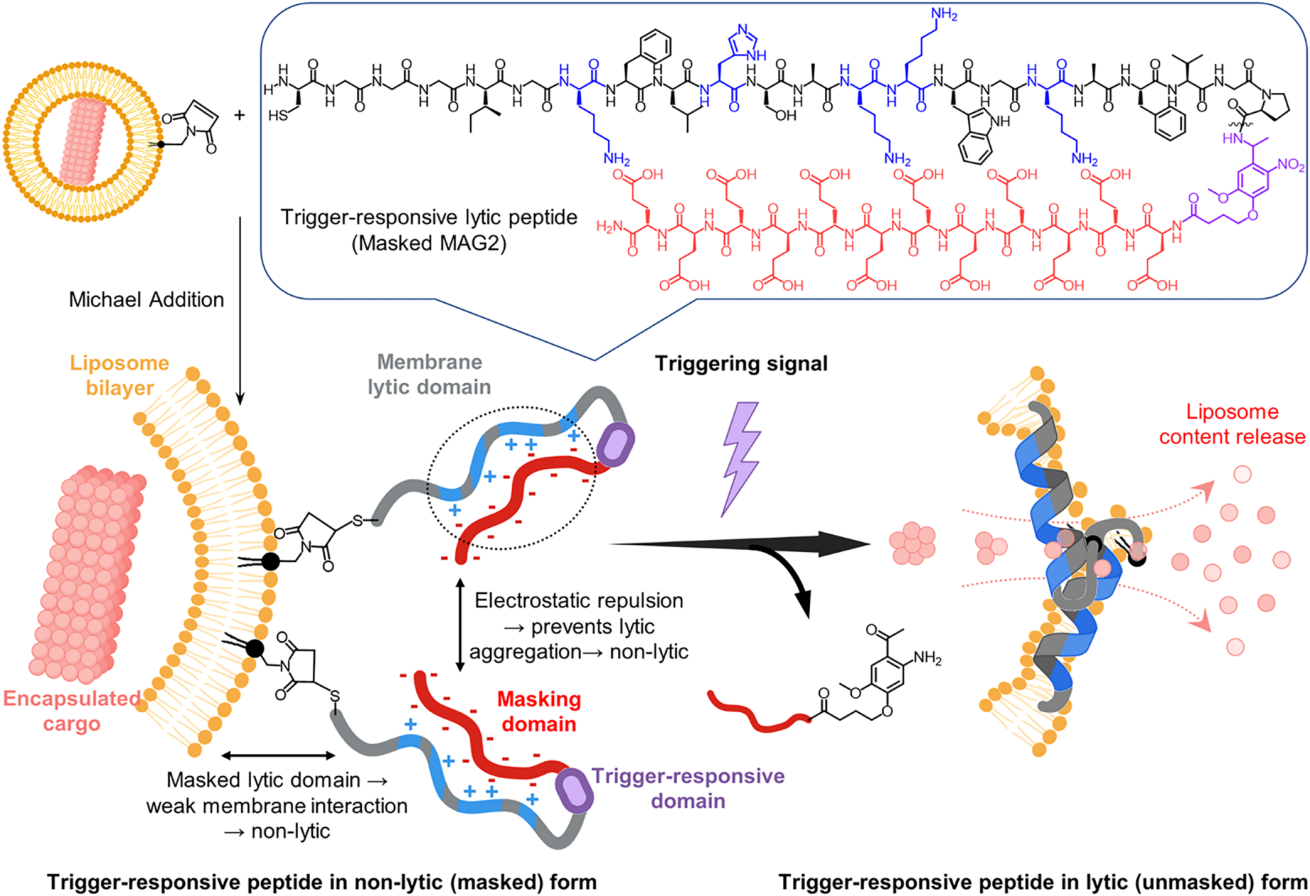
Schematic of trigger-responsive peptidyl liposomes. Trigger-responsive membrane-lytic peptides consist of an AMP backbone (MAG2 as an example), a light-cleavable linker, and a polyglutamate (12E) masking domain. AMPs membrane lyticity needs to be fully suppressible by the masking domain to prevent premature release. Upon UV irradiation, the ortho-nitrobenzyl linker undergoes intramolecular hydrogen abstraction followed by aci-nitro rearrangement, leading to cleavage of the benzylic C–N bond adjacent to proline and form 12E-ortho-amino aryl ketone.^30^ Once the masking domain is photolytically removed, the membrane lyticity of MAG2 is restored and drives the liposomal content release.

To establish an optimal trigger-responsive platform, we selected nine cationic amphipathic AMPs differing in origin and many other physicochemical properties as candidate backbones (Table S1). The peptide sequences are listed in the table in Figure 2, and their helical wheel diagrams—representing their membrane-lytic secondary structures—are shown in Figure S1. By comparing the release profiles of masked and unmasked peptides, we identified the AMP exhibiting the greatest difference in release behavior and employed it as the backbone for constructing a masked peptidyl liposome to assess latent encapsulation stability and trigger-release performance. Furthermore, peptide–membrane interactions in the covalently conjugated (unary) form were visualized by cryo-electron microscopy (cryo-EM) to gain structural insights into the liposome–peptide interface.

**Figure 2:**
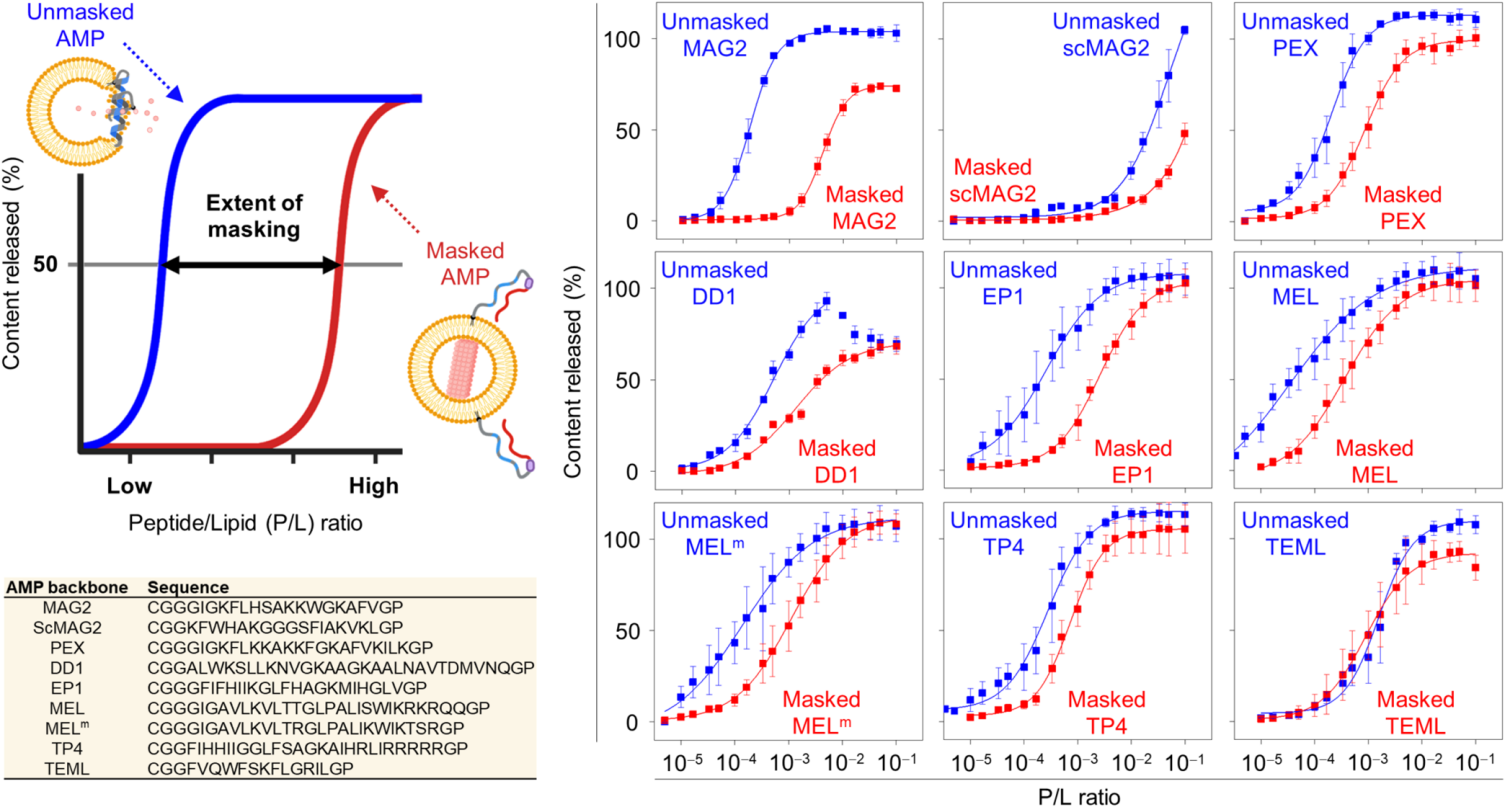
Selection of AMP backbones based on masking efficiency measured by a covalent titration assay. Unmasked (filled blue squares) and masked (filled red squares) AMPs were covalently conjugated to liposomes, and peptide-induced release profiles were plotted as a function of the peptide-to-lipid (P/L) ratio. Masking efficiency was quantified as the ratio of [P/L]_50_ (masked) to [P/L]_50_ (unmasked), where [P/L]_50_ denotes the P/L ratio required to achieve a 50% content release. The masking efficiencies obtained for MAG2, scMAG2, PEX, DD1, EP1, MEL, MEL^m^, TP4, and TEML were 34.8-, 4.9-, 5.4-, 7.9-, 11.5-, 9.0-, 7.1-, 3.3-, and 0.8-fold, respectively. Data are presented as mean ± SD (*n* = 4). Sequences of unmasked AMPs are given in the table within.

## Results and Discussion

### Design Rationales of Trigger-Responsive Peptides

In our design, (1) we employed linear cationic amphiphilic membrane-lytic AMPs as the backbone, with an N-terminus Cys–Gly–Gly tripeptide linker for conjugation to liposomes containing a maleimido anchoring lipid, (2) a β-turn Gly–Pro dipeptide with a light-cleavable linker attached to the C-terminus of the AMP, and (3) a dodecaglutamate (12E) attached to the C-terminus of the photolabile linker (length selection of polyglutamate is shown in Figure S2). The AMP masking using 12E has two reasons. First, intramolecular electrostatic interactions between AMP and 12E, reinforced by the β-turned Gly–Pro dipeptide, prevent AMP from adopting a membrane-lytic amphiphilic helix, thereby reducing its membrane lyticity. Second, intermolecular electrostatic repulsion between 12E further hinders lateral AMP aggregation, preventing peptide-induced membrane disruption. After 12E was photolytically removed, only AMP remained conjugated on the liposome to induce content release. Light trigger was selected in this pioneering peptide–membrane unary system for a rapid and straightforward unmasking strategy without side reactions or unexpected complexities, allowing us to focus on AMP selection and peptide–membrane interaction in unary systems. The rapid and precise photounmasking was showcased by 12E-masked magainin 2 (MAG2) (Figure S3).

### Screening AMP Backbones for Effective Maskability and Membrane Lyticity

To prevent severe premature release in peptidyl liposomes, a comprehensive understanding of AMP maskability is essential for selecting an appropriate AMP backbone to develop trigger-responsive peptidyl liposomes with stable encapsulation during the signal-waiting stage.^18^ Both unmasked and masked forms of selected AMPs were synthesized and characterized (Table S2). To evaluate membrane-lytic activity of peptides conjugated to liposomes, we developed a “covalent titration assay” plotting liposome release curves against varying peptide conjugation amounts, with membrane lyticity quantified by the peptide-to-lipid (P/L) ratio needed for 50% release ([P/L]_50_) (Figure 2). Masking efficiency was calculated as [P/L]_50_^masked^/[P/L]_50_^unmasked^. All peptides showed strong membrane lyticity in their unmasked states, including MAG2, thought to be inactive against zwitterionic membranes, except for the negative control, scrambled magainin 2 (scMAG2). This suggests that zwitterionic membrane-inert peptides in binary systems, such as MAG2, can become highly membrane-lytic when conjugated, possibly due to a proximity effect. Surprisingly, MAG2 displayed remarkable maskability with a 34.8-fold increase in [P/L]_50_, outperforming other peptides. EP1 and DD1 showed masking efficiencies of 11.5- and 7.9-fold, respectively, while others showed little effect. This remarkable right-shift in the membrane-lyticity curve of masked MAG2 demonstrates that previous studies have not identified a suitably maskable AMP backbone. Here, we exploit the exceptional maskability of MAG2 to construct trigger-responsive peptidyl liposomes (**masked MAG2-Lp**).

### Trigger-Responsive Liposomal Release Analysis

Following the synthesis of trigger-responsive masked MAG2, we evaluated P/L ratios from 1/1200 to 1/300 to determine the optimal peptide surface substitution level. All liposomes maintained excellent encapsulation stability with negligible premature leakage. A P/L ratio of 1/300 yielded ∼80% trigger-induced release (Figure S4), and peptide conjugation efficiency was estimated to be approximately 80% by size exclusion chromatography (Figure S5), which was selected for further experiments. As expected, the negative control **masked scMAG2-Lp** showed minimal trigger release, indicating that it is the AMP backbone sequence, rather than amino acid composition, that governs liposomal release. Time-dependent zeta potential measurements of peptidyl liposomes over irradiation time clearly showed that the 12E masking domain was photolytically removed within 1 min for both **masked MAG2-Lp** and **masked scMAG2-Lp** (Figure 3a). Time-dependent trigger-release measurements showed **masked MAG2-Lp** reached a release plateau around 80% within 0.5–1 h at 37 °C, while the release of **masked scMAG2-Lp** is insignificant (Figures 3b and S6, respectively). In the absence of the trigger, the peptidyl liposome showed no release, similar to another control where the liposome did not conjugate with peptide (**Lp**), confirming the excellent encapsulation stability and trigger-release performance of **masked MAG2-Lp**. Particle size and zeta potential measurements of **masked MAG2-Lp** are shown in Figure 3c, and the results for all other liposome controls are listed in Table S3. Furthermore, the size and zeta potential measurements for **masked MAG2-Lp** across a range of P/L ratios (1/600 to 1/1200) are detailed in Table S4. Temperature-dependent trigger-release studies revealed that **masked MAG2-Lp** responds optimally between 37 and 46 °C (Figure S7). Even at 46 °C, all liposomes including **masked MAG2-Lp** show excellent encapsulation stability during the signal-waiting stage. This trend is also held in serum-rich environments at 37 °C (Figure S8), indicating potential for in vivo applications if the photoresponsive domain is replaced with disease-associated trigger domains. Calcein influx analysis of giant unilamellar vesicles (GUVs) revealed that MAG2-induced membrane permeability decayed with a half-lifetime of ∼33 min (Figure S9), indicating that peptide-induced membrane defects persist for about an hour. This duration, far longer than that of freely acting magainin 2 in binary systems,^31^ suggests more sustained peptide–membrane interactions when MAG2 is surface-anchored. Consistently, doxorubicin release plateaued at ∼80% within 0.5–1 h after photoactivation. Together, both results define a unified temporal window in which peptide unmasking, aggregation, and membrane remodeling proceed over hour-scale kinetics governing the triggered release process.

**Figure 3:**
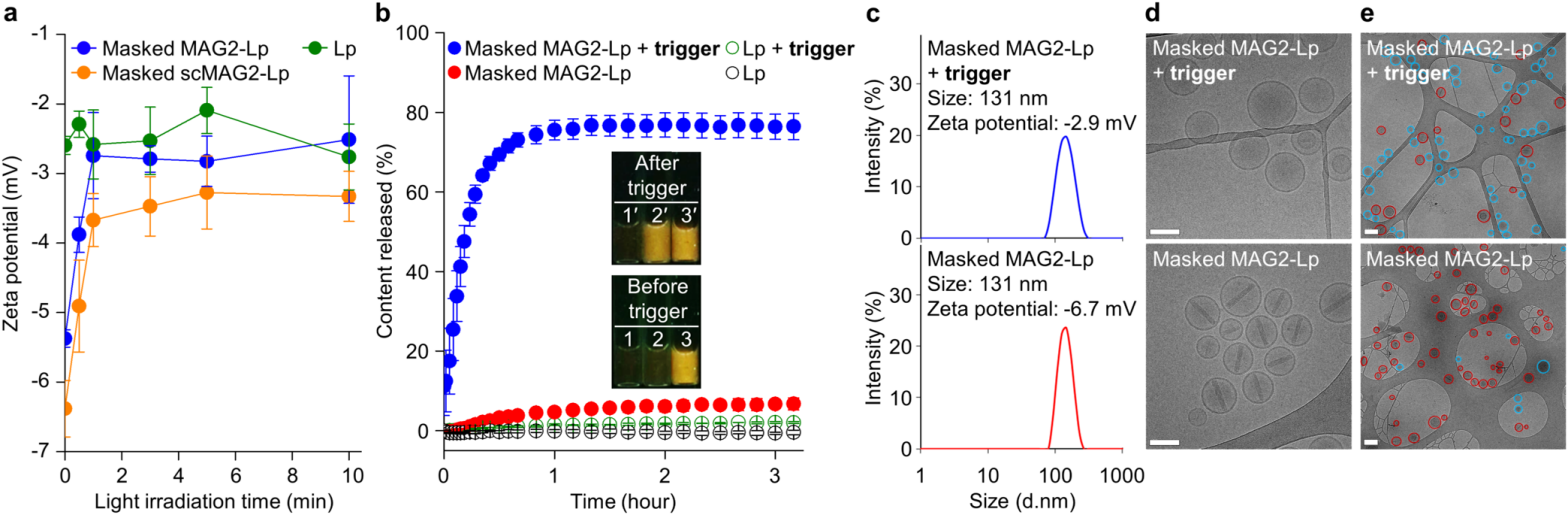
(a) ζ-potential of liposomes with different light irradiation durations (0–10 min), followed by incubation at 37 °C for 1 h. Data are presented as mean ± SD (*n* = 3). (b) Trigger-release profiles of **masked MAG2-Lp** and control liposome **Lp** with or without a trigger at 37 °C. Data are presented as mean ± SD (*n* = 3). Inset: Before (bottom) and after trigger (top) of (1 and 1′) **Lp**, (2 and 2′) **masked MAG2-Lp**, and (3 and 3′) Triton X-100 disrupted **masked MAG2-Lp**. (c) Size and zeta potential of **masked MAG2-Lp** with or without trigger. (d) Representative cryo-EM micrographs of **masked MAG2-Lp** with or without trigger at a magnification of 50,000×. Scale bar: 100 nm. (e) Representative cryo-EM micrographs of **masked MAG2-Lp** with or without trigger at a magnification of 5000×. Doxorubicin-entrapped liposomes are circled in red, and empty liposomes are circled in blue. Empty liposome percentages of **masked MAG2-Lp** with and without trigger are 75% (131/175) and 9% (13/140), respectively. Scale bar: 200 nm.

Cryo-EM can visualize, at the single liposome level, the extent doxorubicin releases. Images of **masked MAG2-Lp** before and after trigger are shown in Figure 3d, and images for all other liposome controls are shown in Figure S10a. Fluorescence assays indicated 80% doxorubicin release, and cryo-EM liposome counting unambiguously showed that ∼20% of liposomes had no release of their contents (Figures 3d and S10b). Notably, the trigger-released liposomes appeared hollow but intact, implying that peptide action involves transient leakage defects or stable pore formation. Liposome particle concentration analysis by dynamic light scattering (DLS) before and after trigger are similar (4.96 × 10^9^ and 5.28 × 10^9^, respectively) without particle size change, also suggesting release is caused by peptide-induced transient membrane defect/pore rather than permeant fragmentation or micellization. Further cryo-EM analysis of peptide–membrane interactions in a mechanistic view will be discussed later.

To evaluate the in vitro efficacy, fluorescence microscopy, flow cytometry, and fluorescence-lifetime imaging microscopy (FLIM) were used to assess the trigger release in KB cells. Fluorescence microscopy showed strong nuclear fluorescence in cells treated with **masked MAG2-Lp** after the trigger, similar to that in free doxorubicin-treated cells, indicating efficient doxorubicin release (Figure 4a). In contrast, negative controls (**masked MAG2-Lp** without trigger, **masked scMAG2-Lp** w/wo trigger, or **Lp** w/wo trigger) showed weak, punctate cytosolic fluorescence, suggesting no trigger release (Figures 4a and S11). Flow cytometry-based doxorubicin/Annexin V-Cy5 staining assay (Figure 4b) revealed significant apoptosis (50.5%) in cells treated with **masked MAG2-Lp** and trigger, comparable to free doxorubicin with trigger (53.5%), whereas minimal apoptosis (14.0% and 12.3%) was seen in cells treated with **masked MAG2-Lp** or control **Lp** with trigger, respectively.

FLIM mapping was employed to analyze the fluorescence lifetime and distinguish whether the doxorubicin fluorescence originated from its encapsulated or released form in treated cells. According to the literature, free doxorubicin in the nucleus has a lifetime of 1.5–2.5 ns,^32–34^ while in the cytosol, it typically has a lifetime of 3.1–4.1 ns.^32,33^ Encapsulated liposomal doxorubicin has low quantum yield, with three coexisting species showing lifetimes of 0.2, 1.0, and 4.5 ns.^35^ In our experiment (Figure 4c), cells treated with **masked MAG2-Lp** followed by trigger release showed strong nuclear (τ = 2.4 ns) and cytosolic fluorescence (τ = 4.2 ns), consistent with free doxorubicin, indicating effective release and uptake. Negative controls (**masked MAG2-Lp** without a trigger and **Lp** without a trigger) showed dim nuclear fluorescence and faint cytosolic puncta (τ = 2.4 ns), suggesting minimal liposome internalization. Very few liposomes uptake causes faint puncta, suggesting that very few liposomes underwent endocytosis. Those internalized liposomes were degraded and released doxorubicin binding to nucleic acids in endosomes/lysosomes (2.4 ns). These results highlight the importance of extracellular drug release at the target tissue, as opposed to relying on cellular uptake, for more effective treatment. The efficacy of liposomal doxorubicin depends on its release efficiency and kinetics.

Conventional liposomal doxorubicin, lacking an efficient release mechanism, has a higher IC_50_ (100 μM) compared to free doxorubicin (IC_50_ of 1 μM),^36^ making it safer but less effective. Dose-dependent cytotoxicity studies with **masked MAG2-Lp**, free doxorubicin, and negative controls (Figure 4d) showed that **masked MAG2-Lp**, after trigger application, had potent toxicity with a low IC_50_ of 2.0 μM, similar to that of free doxorubicin (IC_50_ of 0.7 μM). Without the trigger, **masked MAG2-Lp** exhibited minimal toxicity with a high IC_50_ of 92.5 μM, comparable to conventional liposomal doxorubicin.^36^ This demonstrates a 46-fold increase in inducible cytotoxicity, with maximal cytotoxicity similar to that of free doxorubicin. In a fixed-dose experiment, cells treated with triggered **masked MAG2-Lp** had comparable efficacy to free doxorubicin, with cell viability dropping below 20% (Figure 4e). During the trigger-waiting stage, cytotoxicity remained low with cell viability above 80%, similar to conventional liposomal doxorubicin. This trigger-responsive feature is essential for minimizing toxicity at unintended sites, where no trigger signal is present.

**Figure 4:**
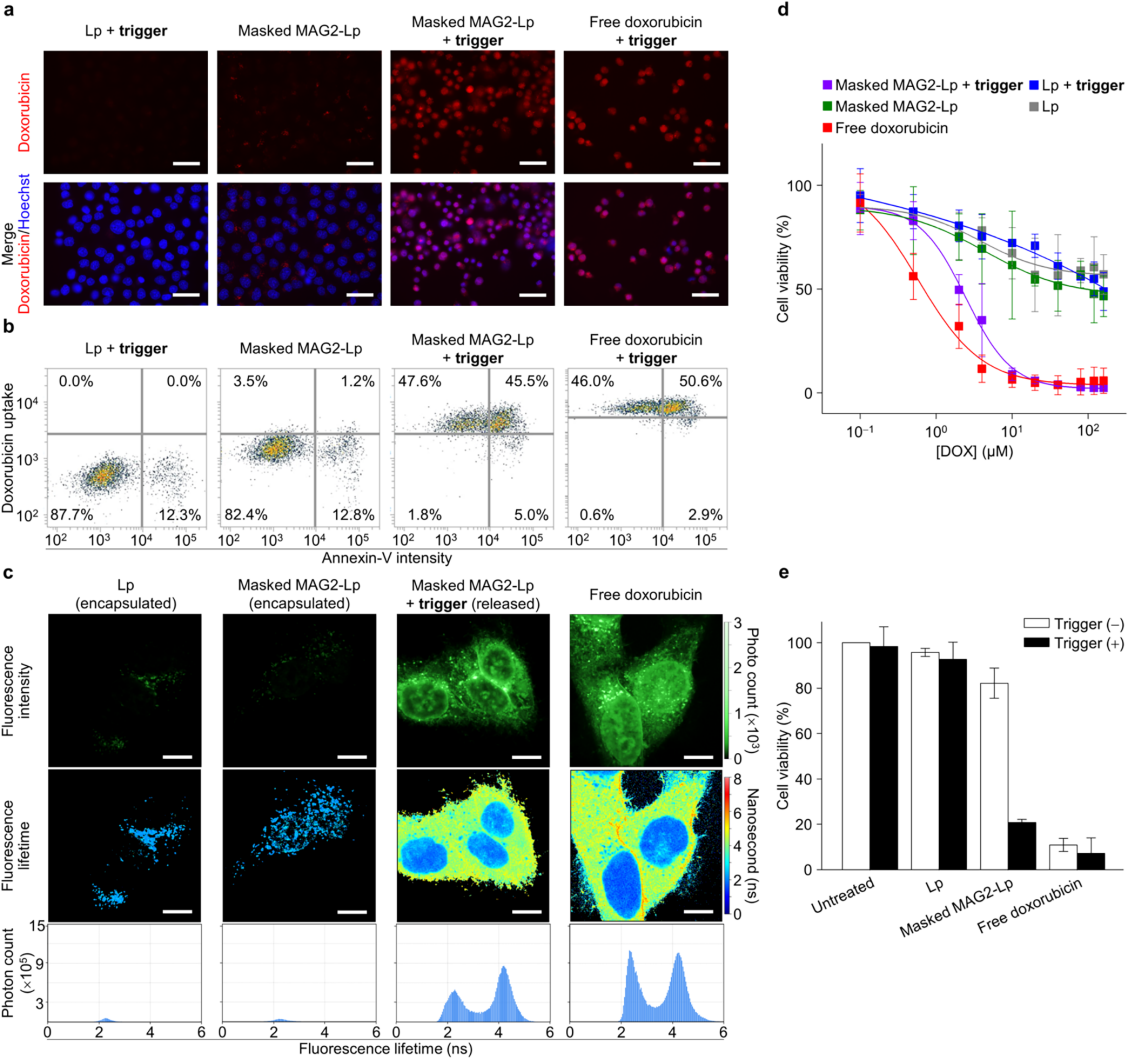
(a) Fluorescence images of KB cells treated with liposomes or free doxorubicin, either with or without a trigger, followed by incubation at 37 °C for 20 h. Merged images show doxorubicin fluorescence (red) and nucleus Hoechst staining (blue). Scale bar: 100 μm. (b) Flow cytometry analysis of cells treated with liposomes or free doxorubicin, either with or without trigger, followed by incubation at 37 °C for 20 h. (c) FLIM images and corresponding photon count histograms of cells treated with liposomes or free doxorubicin, either with or without trigger, followed by incubation at 37 °C for 20 h. Doxorubicin fluorescence intensity is indicated by green brightness. Scale bar: 20 μm. Doxorubicin fluorescence lifetime is represented from blue (short) to red (long). The corresponding lifetime histograms for the nucleus and cytoplasm were 2.4 and 4.2 ns, respectively. (d) Cell viability after treatment with different concentrations of liposomal or free doxorubicin, either with or without trigger, determined by MTT assay. Data are presented as mean ± SD (*n* = 3). (e) Cell viability after treatment with 12.5 μM liposomal or free doxorubicin, either with or without trigger, measured by the MTT assay. Data are presented as mean ± SD (*n* = 2).

### Mechanism Study of Unary Peptide–Membrane Interactions

The 12E masking domain plays a critical role in suppressing the membrane-induced helicity of MAG2 in its conjugated state. Circular dichroism (CD) spectra (Figure 5a) showed that unconjugated MAG2 remained largely unstructured in PBS (6%) and liposome suspensions (7%). In contrast, liposome-conjugated or sodium dodecyl sulfate (SDS)-solubilized MAG2 displayed pronounced helicity (43% and 50%), reflecting strong peptide–membrane interactions characteristic of unary systems, unlike those observed in conventional binary peptide–membrane models.^37^ When masked with 12E, MAG2 retained a low helicity despite potential electrostatic competition from membranes or detergents, confirming effective masking. Specifically, 12E reduced MAG2’s helicity from 43% to 17% in the conjugated state and from 50% to 22% in SDS solution. These results demonstrate that 12E efficiently suppresses the amphiphilic folding of membrane-anchored MAG2, ensuring stable encapsulation without spontaneous leakage.

**Figure 5:**
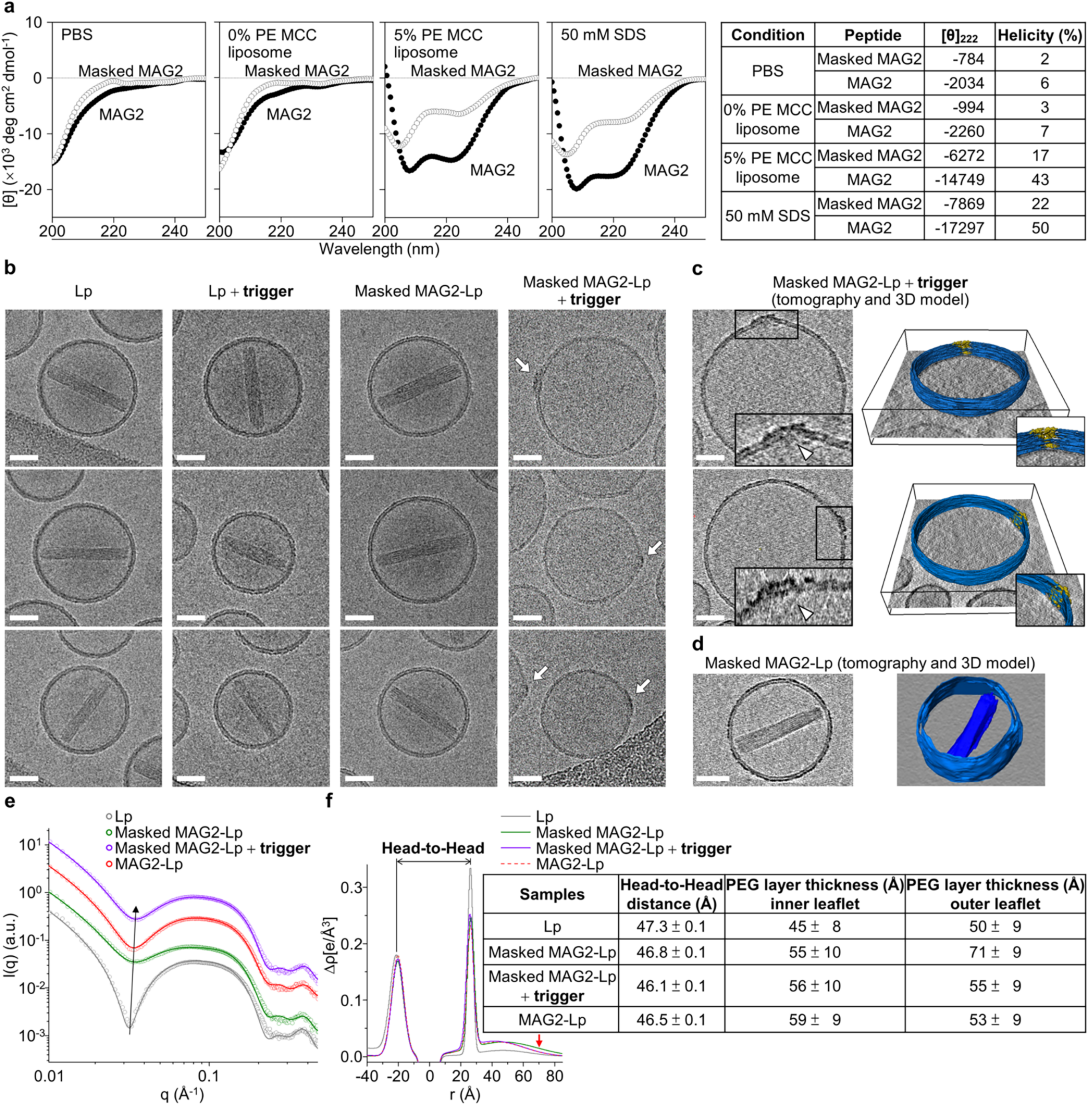
(a) CD spectra of peptides under various conditions. 12 μM masked MAG2 (open black circles) or MAG2 (filled black circles) was in PBS, either not conjugated to liposomes (without thiol-reactive anchoring lipid PE MCC) or conjugated to liposomes (with 5% PE MCC), and without any liposomes but in 50 mM sodium dodecyl sulfate (SDS). The dashed gray line indicates the zero ellipticity point. The [θ]_222_ values and helicity of peptides in different environments are shown in the adjacent table. (b) Positive staining cryo-EM images of liposomes (triplicate). White arrows indicate UA-stainable peptide clusters. Scale bar: 50 nm. (c) Cryo-ET images of **masked MAG2-Lp** after the trigger (duplicate). White triangles indicate peptide aggregates, and the tomography images (insert: magnified peptide aggregation region) reveal that these aggregates caused localized membrane defects. Liposome membranes are shown in blue, and peptide aggregations in yellow. Scale bar: 50 nm. (d) Cryo-ET images of **masked MAG2-Lp** before trigger, and the tomography image reveals the clear lipid bilayer (blue) with encapsulated doxorubicin (navy blue). Scale bar: 50 nm. (e) SAXS profiles of doxorubicin-free liposomes. The black arrow marks the progressive high-q shift of the characteristic SAXS hump after the conjugation of peptides. (f) Corresponding electron density (ED) profiles fitted from the SAXS data, where the head-to-head (black double arrow) distance represents the membrane thickness; the red arrow indicates the reduction of the masked MAG2 to the outer leaflet of the liposome after UV triggering (the full-scale ED profiles are shown in Figure S13). Head-to-head distance and PEG-layer thicknesses on the inner and outer leaflets of the liposomes are summarized in the adjacent table.

Conventional binary peptide–membrane studies have shown that nonhemolytic AMPs like magainin 2 disrupt only negatively charged membranes—not zwitterionic ones such as clinical liposomes—via two mechanisms: toroidal pore formation (by neutron diffraction with both peptide and membrane resolved)^25,38^ and carpet/micellization^25,39,40^ (by cryo-EM without direct peptide visualization).^41,42^ Notably, both require extremely high P/L ratios (∼1/25).

For the unary peptidyl liposome system, it is crucial to elucidate the distinct peptide–membrane interaction mode for achieving effective masking and trigger release. We discovered the MAG2 is very effective for liposomal release upon membrane conjugation; therefore, our unary peptide-to-membrane interaction mechanism studies start from a low P/L ratio of 1/300. To visualize the peptide behavior directly, uranyl acetate (UA) for peptide staining was applied for cryo-EM analysis. Images of **masked MAG2-Lp**, after 12E trigger removal, revealed that peptide aggregates were UA-stained and localized to small, confined patches on the liposomal membrane, indicating localized rather than global membrane disruption underlies doxorubicin release at P/L ratio of 1/300 (Figure 5b). In contrast, all negative controls—including untriggered **masked MAG2-Lp**, liposomes alone, and triggered liposomes without peptide—retained intact morphology, showing neither peptide aggregation nor drug release. These findings indicate that photolytic removal of the 12E masking domain effectively triggers peptide aggregation, which in turn disrupts the membrane to release encapsulated contents.

Quantitative cryo-EM analysis further confirmed that among 183 total trigger-applied **masked MAG2-Lp**, 151 of them were emptied, with 54 of the empty liposomes exhibiting visible peptide aggregates (Table S5). Considering the two-dimensional (2D) projection limitation of cryo-EM—where aggregates located on the upper or lower liposome surfaces might not be visible—this represents a notably high aggregate-to-liposome ratio. In contrast, negative controls showed insignificant emptied liposomes without visible aggregates, reinforcing that photoinduced peptide aggregation is the primary cause of membrane defect formation and content release. Moreover, distinct peptide–membrane interaction modes at different P/L ratios were revealed. At P/L ratio = 1/300, lateral peptide aggregates confined to the outer leaflet suggested a carpet-like mechanism (white arrow, Figure 5b). At P/L ratio = 1/100, the coexistence of peptide aggregates (white arrow) and membrane fragmentation (black arrow) indicated a transition toward micellization (Figure S12).

Cryo-electron tomography (Cryo-ET) provided a 3D image of trigger-activated **masked MAG2-Lp**, showing peptide aggregation-induced membrane defects with encapsulated doxorubicin released (Figure 5c). In contrast, **masked MAG2-Lp** without a trigger showed intact bilayers and doxorubicin well-encapsulated (Figure 5d). These findings indicate that unmasked MAG2 lateral aggregates on liposomes cause membrane defects and release content.

To further elucidate the structural impact of peptide–membrane interactions at the liposomal interface, small-angle X-ray scattering (SAXS) was employed to examine the bilayer architecture and surface organization of doxorubicin-free **masked MAG2-Lp** before and after trigger activation. The broad humps (centered at the scattering vector *q* ∼ 0.08 Å^–1^) of the SAXS profiles showed asymmetric broadening due to the asymmetric conjugation of masked MAG2 or unmasked MAG2 to the outer leaflet; these conjugations also result in a slight shift of the first minimum of the scattering profiles (*q* ∼ 0.03 Å^–1^) toward higher-*q* regions, leading to a reduction of the head-to-head distance (or membrane thickness) as well (Figure 5e).^43^ To obtain quantitative information on membrane thickness, representative electron density profiles were fitted from the SAXS data (Figure 5f), employing a flat asymmetric model known as the five-layer model.^44^ The head-to-head distance between two phosphate peaks (representing membrane thickness)^45^ and the PEG-layer thickness of liposomes were calculated and listed in the table of Figure 5f. Trigger-applied **masked MAG2-Lp** showed a thinner membrane (46.1 Å) compared to that of **masked MAG2-Lp** without trigger (46.8 Å). Although the overall membrane thinning appears modest, the SAXS-derived thickness reflects an average over the entire vesicle population. Therefore, a decrease of merely ∼0.7 Å, resulting from only ∼0.5% of peptide-anchored lipids, implies pronounced local thinning within peptide-enriched patches (as seen in Figure 5b,c), which is averaged out by the large unaffected membrane area. This phenomenon is consistent with previous observations in binary peptide–membrane systems, where magainin 2 disrupts lipid packing and induces membrane thinning in the negatively charged membrane system.^45,46^ More interestingly, SAXS detected significant PEG-layer structural variation in the outer membrane leaflet where peptide conjugation interfaces—a feature beyond the resolution of cryo-EM. Compared with **Lp**, whose outer PEG-layer thickness is 50 Å, **masked MAG2-Lp** exhibited an expanded outer PEG layer of 71 Å (21 Å expansion), attributable to steric hindrance from free laterally diffusive, masked MAG2. After trigger application, unmasked MAG2 aggregated and localized within confined surfaces, causing most of the PEG layer to recompact to 55 Å (16 Å compaction). This phenomenon may be explained by the outward PEG layer being mechanically supported and thickened by freely diffusing masked peptides, which, upon unmasking, lose their lateral mobility and undergo aggregation, thereby causing the PEG layer to collapse—a multistep transition that cryo-EM was able to visualize only in its later, post-trigger-activation stage. Together, these complementary findings delineate the distinct behaviors of MAG2 at the liposomal interface before and after unmasking. In the signal-waiting state, freely lateral-diffused, masked MAG2 maintains membrane-inertness, retains membrane integrity, and expands/thickens the PEG layer, whereas in the trigger-unmasked state, MAG2 aggregates and disrupts membrane integrity in a very confined area, leading to PEG-layer recompaction.

## Conclusions

This study establishes a unified mechanistic framework for designing encapsulation-stable, yet trigger-responsive peptidyl liposomes by elucidating how membrane-anchored peptides engage lipid bilayers in the unary (covalently conjugated) state. Historically, premature release upon peptide conjugation has hindered the progress, largely due to limited understanding of tethered peptide–membrane interaction—particularly proximity effect, lateral diffusion, and membrane selectivity of peptides. Through our screening, we identified the zwitterionic membrane-inert peptide MAG2 as an optimal backbone for engineering trigger-responsive membrane-lytic peptides that need to be covalently anchored onto zwitterionic liposomes.

Cryo-EM/cryo-ET and SAXS together reveal previously uncharacterized structural transitions in unary peptide–liposome systems. SAXS captures the outer leaflet PEG-layer expansion (∼21 Å) upon peptide conjugation and its collapse (∼16 Å) after peptide unmasking, while cryo-EM/cryo-ET resolves the emergence of lateral peptide aggregates following trigger-unmasking. These observations indicate that masked MAG2 freely lateral-diffuses and supports an extended “stand-up” PEG conformation, whereas unmasked MAG2 localizes and aggregates on the membrane, induces partial membrane thinning, and is unable to support the PEG layer, leading to its collapse. Combined with doxorubicin release assays, CD, calcein influx, FLIM/fluorescence imaging, and MTT cytotoxicity analyses, our results delineate a hierarchical sequence of events: masked peptide–membrane conjugation with stable encapsulation, lateral-diffusion-driven PEG-layer expansion, peptide unmasking, aggregation-mediated membrane thinning and PEG-layer collapse, transient defect formation, subcellular level of liposomal release and distribution, and trigger-induced cytotoxicity.

This work provides three conceptual advances. First, MAG2 is identified as the only AMP backbone to date that remains fully maskable when covalently conjugated, enabling truly trigger-responsive unary systems—an ability likely rooted in its zwitterionic membrane-inert character. Second, MAG2, long considered inactive toward zwitterionic membranes in binary systems, is found to be strongly membrane-lytic when surface-anchored. Third, the conjugated masked MAG2 can diffuse freely on the membrane, whereas trigger-unmasked MAG2 undergoes localized lateral aggregation and transiently permeabilizes the membrane. Together, these findings in unary peptide–membrane systems redefine peptide–membrane interaction paradigms and offer a new conceptual path for achieving both stable encapsulation and effective on-demand activation in unary peptidyl liposomes.

Overall, this work provides a generalizable foundation for the rational design of unary trigger-responsive peptidyl liposomal vesicles, establishing principles that will guide the future development of peptide-engineered, mechanism-driven, programmable liposome delivery systems.

## Materials and Methods

All other chemicals were obtained from Sigma-Aldrich unless otherwise specified. 1,2-Distearoyl-*sn*-glycero-3-phosphocholine (DSPC), 1,2-distearoyl-*sn*-glycero-3-phosphoethanolamine-*N*-[methoxy­(polyethylene glycol)-2000] (ammonium salt) (DSPE-PEG2000), 1,2-dipalmitoyl-*sn*-glycero-3-phosphoethanolamine-*N*-[4-(p-maleimidomethyl)­cyclohexane-carboxamide] (sodium salt) (16:0 PE MCC), and 1,2-dioleoyl-*sn*-glycero-3-phosphoethanolamine-*N*-[4-(p-maleimidomethyl)­cyclohexane-carboxamide] (sodium salt) (18:1 PE MCC) were purchased from Avanti Polar Lipids (Alabaster, AL).

### Liposome Preparation

Generally, liposomes were prepared by the thin-film rehydration method, followed by freeze–thaw and extrusion techniques. A lipid film was formed by rotary evaporation of DSPC/Cholesterol/DSPE-PEG2000/16:0 PE MCC/18:1 PE MCC (molar ratio = 45:50:5:0.75:0.25) from chloroform and dried overnight in a vacuum desiccator. The lipid (approximately 12 mg, 17.6 μmol) film was hydrated in 1 mL of a solution containing 250 mM ammonium sulfate at room temperature. The suspension was subjected to ten freeze–thaw cycles in liquid nitrogen/60 °C water bath and subsequently extruded 21 times using a Mini Extruder (Avanti Polar Lipids) through a track-etch 100 nm polycarbonate membrane at 60 °C to obtain uniform-sized vesicles. The ammonium sulfate solutions were then exchanged with a 150 mM NaCl solution using Sepharose CL-4B. Doxorubicin was actively loaded at a drug-to-lipid ratio of 1:10 and incubated at 65 °C for 40 min. Finally, the doxorubicin-encapsulated liposome was separated from trace amount of nonencapsulated doxorubicin using Sepharose CL-4B equilibrated with tricine buffer (50 mM tricine, 100 mM NaCl, pH 7.5). All liposomes were prepared by using the above method unless otherwise specified. For the covalent titration assay, liposomes were prepared using DSPC/Cholesterol/DSPE-PEG2000/18:1 PE MCC (44:50:5:1); for CD experiments, liposomes were prepared using DSPC/Cholesterol/DSPE-PEG2000/18:1 PE MCC (45:50:5:0 or 40:50:5:5), the lipid films were hydrated with PBS (Gibco), and the resulting liposome suspensions were extruded 21 times through 50 nm polycarbonate filters at 60 °C; for SAXS experiments, doxorubicin-free liposomes were prepared using DSPC/Cholesterol/DSPE-PEG2000/18:1 PE MCC (44:50:5:1), the lipid films were hydrated with tricine buffer, and the resulting liposome suspensions were extruded 21 times through 50 nm polycarbonate filters at 60 °C.

### Covalent Titration Assay

To measure the membrane lyticity of peptides in the conjugation stage, peptides were titrated and reacted with liposomes at different P/L ratios, and the liposomal doxorubicin release was measured. Peptides (0.2 mM) were first reduced with TCEP (0.4 mM) in tricine buffer at room temperature for 15 min. Subsequently, peptide solutions were diluted accordingly, added to the liposome with 15 μM apparent doxorubicin concentration at different P/L ratios, and incubated at 37 °C for 2 h. The fluorescence intensity of doxorubicin was then measured at an excitation wavelength of 490 nm and an emission wavelength of 590 nm by using an EnSpire multimode plate reader (PerkinElmer). The percentage of doxorubicin release was calculated as follows: Doxorubicin released (%) = 100 × (*I* – *I*_0_)/(*I*_max_ – *I*_0_), where *I* represents the fluorescence intensity of peptide-conjugated liposomes, *I*_0_ is the initial liposome fluorescence intensity without peptide addition, and *I*_max_ is the maximum fluorescence intensity by adding Triton X-100 (final concentration of 1% (v/v)) and incubated at 70 °C for 2 min.

### Peptidyl Liposome Preparation

To conjugate the peptide on the liposome surface, peptides (0.2 mM) were reduced with TCEP (0.4 mM) in tricine buffer at room temperature for 15 min to free all disulfided cysteine before they were added to liposome solutions. Subsequently, peptide solutions were added to the liposome (final concentration of lipids was 0.675 mM) at a P/L ratio of 1/300 and incubated on a reciprocal shaker at 120 rpm and 37 °C for 1 h. The peptidyl liposomes were size-excluded from unreacted peptides using Sepharose CL-4B preequilibrated with tricine buffer.

### Liposome Release Assay

Liposome solutions were prepared at an apparent concentration of 15 μM doxorubicin. Liposome solutions were either kept in the dark or irradiated using UVP Mineralight UV Lamps (UVL-225D) (365 nm, 3 mW/cm^2^ for 10 min) and then incubated at 37 °C for 1 h to complete the release.

### Cytosolic Delivery of Doxorubicin Estimation by Microscopy and Flow Cytometry

To visualize fluorescence of doxorubicin in cells delivered by peptidyl liposomes before or after triggering the signal, KB cells (1.1 × 10^4^ cells per well) were seeded on a 96-well plate (PerkinElmer) in DMEM (Gibco) with 10% fetal bovine serum (FBS) (Biological Industries) and 1% penicillin/streptomycin (PS) (Biological Industries) for 20 h. The medium was replaced by fresh MEM (Gibco) for 30 min incubation, then replaced by MEM containing either liposomes or free doxorubicin (12.5 μM), and incubated either in the dark or irradiated by 365 nm light at 3 mW/cm^2^ for 4 min, followed by further incubation at 37 °C for 20 h. Fresh MEM with 10% FBS was replaced for imaging. Live cell nuclei were stained with 10 mg/mL Hoechst 33342 at 1:2000 dilution (ThermoFisher) for 20 min. Cellular uptake of doxorubicin was imaged using an Olympus IX-71 microscope equipped with 40× objective lens and an RT3 color CCD system.

To statistically correlate cellular doxorubicin uptake and cell apoptosis, KB cells (2 × 10^5^ cells per well) were seeded on a 12-well plate (JET BIOFIL) in DMEM with 10% FBS and 1% PS for 20 h. The medium was replaced by fresh MEM for 30 min of incubation, then replaced by MEM containing either liposomes or free doxorubicin (12.5 μM), and incubated either in the dark or irradiated by 365 nm light at 3 mW/cm^2^ for 4 min, followed by further incubation at 37 °C for 20 h. After 20 h of postincubation, the suspended and trypsinized cells were collected by 300×*g* centrifugation, and the cell pellet was resuspended in Annexin V-Cy5 binding buffer (BioVison). The apoptotic phosphatidyl-serine exposure of cells was stained by 5 μL of ready-to-use Annexin V-Cy5 solution (BioVison) in the dark for 5 min. Cellular doxorubicin uptake and cell apoptosis were analyzed with 1 × 10^4^ cell counts using an Attune NxT – 14 color analyzer (ThermoFisher Scientific).

### Using FLIM to Assess Doxorubicin Encapsulation and Release

We applied Q2 FastFLIM system from ISS company (http://www.iss.com/) and Nikon Ti-U inverted microscope with submicrometer automatic controlled XYZ stage. A XY set of Galvo mirrors was used for the nanoposition control of the FLIM image. A water-immersion objective (Nikon Plan Apo 60×/numerical aperture (NA) 1.2) mounted on a piezodevice was applied. The system equipped with 488 nm (5 mW) subnanosecond modulated pulsed laser at the fundamental frequency of 20 MHz was controlled by ISS VistaVision software, which was used for doxorubicin excitation sources. The excitation wavelength was connected by an optical fiber and a band-pass filter to improve wavelength selection. Fluorescence emission from the sample went through a band-pass filter (FF05-500/25-25, Semrock) before being sent to the confocal unit with a GaAs photomultiplier tube (PMT) detector (Hamamatsu, H7422P-40).

A standard solution (10 μM fluorescein for 4 ns lifetime) was used to calibrate a laser scanning confocal nanoscope (Q2 system, ISS). KB cells (1.1 × 10^4^ cells per well) were seeded on a 96-well plate in DMEM with 10% FBS and 1% PS for 20 h. The medium was replaced by a fresh MEM (Gibco) for 30 min incubation, then replaced by MEM containing either liposomes or free doxorubicin (12.5 μM), and incubated either in the dark or irradiated by 365 nm light at 3 mW/cm^2^ for 4 min, followed by further incubation at 37 °C for 20 h. Fresh MEM with 10% FBS was replaced for imaging. Then, the fluorescence and lifetime of doxorubicin were acquired using a 60× water-immersion objective lens on a Q2 system.

### Cell Viability Assay

KB cells (1.1 × 10^4^ cells per well) were seeded on a 96-well plate in DMEM with 10% FBS and 1% PS for 20 h. The medium was replaced by a fresh MEM for 30 min incubation, then replaced by MEM containing either liposomes or free doxorubicin (12.5 μM), and incubated either in the dark or irradiated by 365 nm light at 3 mW/cm^2^ for 4 min, followed by further incubation at 37 °C for 20 h. Fresh MEM with 10% FBS was replaced to incubate cells for another 20 h to assess the toxicity of liposomal doxorubicin. The medium was then replaced with 220 μL of fresh MEM containing 20 μL of MTT stock solutions (5 mg/mL in PBS) and incubated at 37 °C for 4 h. 170 μL of culture medium was then removed, and 200 μL of DMSO was added to solubilize the formazan. The plate was kept on a reciprocal shaker with 120 rpm at 37 °C for 10 min. The absorbance of formazan in DMSO at 540 nm was measured by a plate reader to estimate the cell viability. The viability was calculated as follows: Cell viability (%) = 100 × (*A* – *A*_0_)/(*A*_max_ – *A*_0_), where *A* represents the absorption of treated cells, *A*_0_ is the absorption of MEM without cells, and *A*_max_ is the absorption of the cells without liposome treatment in MEM (maximum viability).

### Using CD Spectroscopy to Study Peptide–Peptide and Peptide–Membrane Interactions

To confirm the secondary structure of peptides in various membranous environments, peptides (0.2 mM) with TCEP (0.4 mM) were incubated in tricine buffer at room temperature for 15 min. The TCEP-reduced peptide solutions (12 μM) were then diluted in PBS, 50 mM SDS solution, 0% PE MCC liposomes (lipid concentration = 1.2 mM), or 5% PE MCC liposomes (lipid concentration = 1.2 mM), maintaining a P/L ratio of 1/100, and incubated at 37 °C for 1 h. The 5% reactive lipid PE MCC in liposomes ensured that all peptides were conjugated to the liposome surface. Circular dichroism (CD) spectra were obtained using a JASCO J-815 spectrometer with a 1 mm quartz optical cuvette (Hellma) at 37 °C, recording wavelengths from 200 to 260 nm. Each spectrum was averaged over ten accumulations. The helicity of the peptides was calculated using the following formula: Helicity (%) = 100 × ([θ]_222_/(−39500 × ((1–2.57)/*n*))),^47^ where [θ]_222_ is the mean residue ellipticity at 222 nm, and *n* represents the number of peptide bonds.

### Using Cryo-EM to Visualize Liposome Morphology, Doxorubicin Release, and Peptide Lateral Aggregation on Membranes

Peptidyl liposomes were stained with uranyl acetate (UA) to vitalize a possible peptide aggregate at a lipid/UA ratio of 1:4 in tricine buffer with a final lipid concentration of 0.7 mM and incubated at 37 °C for 30 min. Briefly, 200-mesh copper grids (HC200-Cu, PELCO) were glow-discharged in an (Ar, O_2_) atmosphere for 15 s on the carbon side. Next, 4 μL of liposome solutions (the final concentration of lipid was 0.7 mM) were pipetted onto grids. Grids were blotted in 100% humidity at 4 °C for 3–4 s and plunge-frozen into liquid ethane cooled by liquid nitrogen using a Vitrobot (FEI, Hillsboro, OR). The specimens were imaged with an FEI Tecnai G2 F20 TWIN Transmission Electron Microscope at 200 keV. Transmission electron microscopy (TEM) imaging was conducted in a bright-field mode at an operating voltage of 200 kV. Images were recorded at a defocus of ∼1.8 μm under low-dose exposures (25–30 e/Å^2^) with a 4k × 4k charge-coupled device camera (Glatan, Pleasanton, CA) at a magnification of 50,000×. Cryo-EM sample preparation and imaging were performed at the Academia Sinica Cryo-EM Facility (Taipei, Taiwan).

### Using Cryo-ET to Visualize Peptide Aggregation-Induced Membrane Defects

The UA-stained liposomes were mixed with 10 nm fiducial gold beads (Ted Pella) and applied onto the freshly glow-discharged Quantifoil R 2/2 200 Holey carbon grid (Quatifoil GmbH, Germany). The grids were then blotted from double sides and plunge-frozen into liquid ethane (precooled by liquid nitrogen) using an FEI Vitrobot Mark IV (ThermoFisher Scientific) and stored in liquid nitrogen until data collection. Cryo-ET was performed on a ThermoFisher Talos Arctica 200 keV field emission gun cryogenic electron microscope equipped with a Falcon III detector (ThermoFisher Scientific) in linear mode using Tomography-4.10.0 software (ThermoFisher Scientific). Each tilt series was collected with a span of 120° (−60° to +60°; bidirectional scheme) with 2° increments accounting for a cumulative dose of around 90 e/Å^2^ at 73,000× magnification (with a corresponding pixel size of 1.4 Å) and a target defocus value was set to −5 μm. Frames of each tilt image were motion-corrected by MotionCor2. The average tilt-series images were then aligned and reconstructed by weighted back-projection with a SIRT-like filter (10 iterations) into tomograms using the IMOD software package. For improving contrast, tomograms were binned to 1k × 1k and denoised by Topaz. Denoised tomograms were segmented by Amira software package (ThermoFisher Scientific) with the “Membrane Enhancement Filter” module and manual refinement. The movies of the segmented model were generated with the Amira software package.

### Using SAXS to Capture Membrane Interface Changes of Liposomes

Liposomes at a P/L ratio of 1/100 (20 mM lipid concentration), either with or without trigger, were submitted for SAXS analysis conducted at TLS 23A SWAXS endstation of the National Synchrotron Radiation Research Center (NSRRC). With a beam of 15.0 keV (wavelength λ = 0.8267 Å) and a sample-to-detector distance of 1830 mm, SAXS data were collected by using a pixel detector Pilatus-1MF of an active area of 169 × 179 mm^2^ and a detector pixel resolution of 172 μm. This single instrument configuration could cover a reasonable *q*-range up to 0.5 Å^–1^ with excellent q-resolution; the scattering wavevector *q* = 4πλ^–1^sinθ, defined by the scattering angle θ and λ, was calibrated with a standard sample of silver behenate. To minimize radiation damages, the 3 mm sample solution cell with thin (12.5 μm) kapton windows (5 mm in diameter) was gently rocked within an area of 1.5 × 1.5 mm^2^ to avoid prolonged spot exposure (ca. 0.5 mm in beam diameter) of the sample solution at RT. Each SAXS profile presented was averaged from ten SAXS data scans (each for 30 s); these ten successive scans could overlap well, suggesting negligible radiation damage effects and no structural transitions involved, and hence a thermodynamically stable system. SAXS data were subtracted with water (or tricine buffer) scattering measured under an identical environment as that used for the liposome solutions with tricine buffer; the data were then corrected for incoming flux, sample thickness, and electronic noise of the detector, as detailed in a previous report.^48,49^

Since PEG chains account for only a small portion (5%) of the entire system, the obtained electron density profile mainly reflects the averaged electron density of both water and PEG chains in the outer and inner regions. Direct determination of the PEG chain length from the peak-to-peak distance in the electron density profile is therefore not feasible. To better estimate the PEG chain conformation, the following relationships were applied: *z*_GP_ = *Z*_HR_ + 2 × (2ln10)^∧0.5^ × σ_HR_ and *L* = (*Z*_PEG_ – *Z*_GP_) + 2 × σ_PEG_.^44^ In these equations, *Z*_GP_ denotes the grating plane, while *Z*_HR_ and σ_HR_ correspond to the position and width of the Gaussian function representing the headgroup region, respectively. The PEG-layer thickness (*L*)—reflecting the chain distribution—is approximated using a Gaussian function with peak position *Z*_PEG_ and width σ_PEG_. The combined use of these two equations provides the estimated dimensions of the outer and inner PEG regions.

### Statistical Analysis

The curve fitting of the covalent titration assay and cell viability were analyzed using a dose–response function in Origin 8. Data are presented as the mean ± SD.

## Supplementary Material





## Notes

Liposomal doxorubicin trigger release from **masked MAG2-Lp**; samples include: (1) **Lp**; (2) trigger-responsive **masked MAG2-Lp**; (3) fully released **masked MAG2-Lp**; and (4) Triton X-100 solubilized **masked MAG2-Lp** (WMV)
Peptide synthesis; characterization data of peptides (MS) and liposomes (DLS, cryo-EM, and cryo-ET); covalent titration assay; trigger release of liposomes; fluorescence images of cells treated with liposomes; and calcine influx GUV assay (PDF)
