# Supplementary material for: Mechanistic Insights into Unary Peptide–Membrane Interactions Enable Stable Encapsulation and Trigger-Responsive Peptidyl Liposomes
Source: J Am Chem Soc. 2026 Feb 18;148(8):8749–60. doi: 10.1021/jacs.5c21158 (PMC12964396; doi:10.1021/jacs.5c21158)
Supplement: Supplementary file 2 [file ja5c21158_si_002.pdf]

Supporting Information

Mechanistic Insights into Unary Peptide-Membrane Interactions Enable Stable Encapsulation and Trigger-Responsive Peptidyl Liposomes

Hua-De Gao[a,b], Jia-Lin Hong[a], Cheng-Bang Jian[a,b,c], Tzu-Ho Chen[a], Ning-Chu Chang[a], Suthasinee Meeroekyai[a,b,d], Ruei-Yu He[a], Yi-Ting Liao[e], Chun-Hsiung Wang[e], Chun-Jen Su[f], U-Ser Jeng[f], Meng-Chiao Ho[e], Yu-Ju Chen[a,b], and Hsien-Ming Lee[a*]

[a]*Institute of Chemistry, Academia Sinica, Taipei 11529, Taiwan*

[b]*Department of Chemistry, National Taiwan University, Taipei 10617, Taiwan*

[c]*Nano Science and Technology Program, Taiwan International Graduate Program, Academia Sinica, Taipei 11529, Taiwan*

[d]*Chemical Biology and Molecular Biophysics Program, Taiwan International Graduate Program, Academia Sinica, Taipei 11529, Taiwan*

[e]*Institute of Biological Chemistry, Academia Sinica, Taipei 11529, Taiwan*

[f]*National Synchrotron Radiation Research Center, Hsinchu 300092, Taiwan.*

[*]Email: leehm@chem.sinica.edu.tw

Materials and methods

Peptide synthesis

Peptides were synthesized on a PS3 peptide synthesizer (Gyros Protein Technologies) using Fmoc solid-phase peptide standard method with NovaSyn®TGR resin (Novabiochem) under 0.1 mmol scale. The coupling chemistry used 0.5 mmol (5 equiv/equiv resin) 2-(6-chloro-1H-benzotriazole-1-yl)-1,1,3,3-tetramethylaminium hexafluoro-phosphate (HCTU, 0.5 mmol) and N-Ethyl-N-(propan-2-yl)propan-2-amine (DIPEA, 1 mmol) for most of the amino acids, except for Fmoc-photolabile linker 0.4 mmol (4 equiv/equiv resin) coupling. Arginine and the first amino acid synthesized after proline were double coupled. Deprotection of Fmoc was done by using 20% v/v piperidine in DMF. Peptides were then cleaved from solid-support in a solution containing 95% Trifluoroacetic acid (TFA), 2.5% Triisopropylsilane (TIPS), and 2.5% H_2O for 90 min to afford crude peptides. Crude peptides were purified by semi-preparative C-18 column using a high-performance liquid chromatography (HPLC) with a specified elution gradient with solvent A: 95% H_2O, 5% acetonitrile, and 0.1% TFA and solvent B: 95% acetonitrile, 5% H_2O, and 0.08% TFA. The mass of peptides was determined by a high-resolution ESI-Q-TOF (Waters, Premier, Manchester, UK) or MALDI-TOF (Bruker, New ultrafleXtremeTM, Bremen, DE) mass.

HPLC-MS analysis of photocleavaged masked MAG2

To verify the high yield and fast kinetics of photolysis and better mass ionized efficacy of peptides, TCEP-reduced masked MAG2 solutions were irradiated by a short burst of UV light (UVATA UPS412 power supplied with a UPH-056 365 nm LED at 60 mW/cm^2 for 2 min) and were analyzed by analytical C-18 column using an HPLC with an elution gradient from 10−90% solvent B in 40 min. It needs to be noted that masked MAG2 (0.2 mM) were incubated with N-hydroxymaleimide (NHM) (0.4 mM) in tricine buffer (50 mM tricine, 100 mM NaCl, pH 7.5) for 15 min to cap the cysteine on the peptide for better ionization in mass. Next, a final concentration of 10 mM DTT solution was added to the peptide solutions and incubated for 30 min. Their mass is determined by LCQ ESI-MS (Thermo Finnigan, San Jose, CA, United States).

Dynamic light scattering (DLS) analysis of liposome size, zeta potential, and particle concentration

Zetasizer Nano ZS instrument (Malvern, United Kingdom) with a He-Ne laser at a wavelength of 633 nm was used for DLS experiments. Liposomes were diluted to a final concentration of 100 μM lipid in tricine buffer (50 mM tricine, 100 mM NaCl, pH 7.5). The size distribution, zeta potential, and particle concentration of the liposome samples were measured at 25°C.

Giant unilamellar vesicles (GUVs) preparation by water-in-oil transfer method

GUVs were prepared and modified as previously reported.[1-2] Briefly, lipids (DSPC/cholesterol /DSPE-PEG2000/18:1 PE MCC/18:1 Liss Rhod PE = 45/50/5/1/0.1) (17.6 μmole) in chloroform were mixed with 500 μL liquid paraffin (mineral oil) by the vortex. The lipid-oil solution was incubated at 90°C with 200 rpm stirring for 30 min to remove chloroform. After chloroform evaporation, 250 μL lipid-oil solution was added to 25 μL inner solution (250 mM sucrose) and kept it at 75°C (for dissolving DSPC). Mixed the above solution by agitating along the tube rack and vortex for 10 s. Incubated the water-in-oil emulsion at 75°C for 10 min to equilibrate the lipid monolayer assembly. Pour 150 μL of outer solution (250 mM glucose) in the tube at 75°C and layer the emulsion mixture on top. Centrifuged the tube at 9000 × g for 1 min, and then liposomes will be formed. Collected the liposome suspension (bottom layer solution) from the tube bottom.

Estimating the duration of transient membrane defects by calcein influx assays[3-4]

To visualize defect formation and subsequent resealing induced by MAG2 conjugation, GUVs (~0.68 mM lipid) were briefly exposed to MAG2 (P/L = 1/300) in the presence of 20 μM external calcein for 1 h. This period encompasses rapid peptide conjugation, immediate membrane disruption, and the slower defect-resealing process. Resealed GUV can retain calcein. After the 1 h incubation, the external calcein concentration was reduced to 10 μM by isotonic dilution, and GUVs that can or cannot retain calcein (indicating that the defect resealed after calcein influx) were imaged by fluorescence microscopy.

In flow cytometry experiment, calcein blocking instead of calcein retaining was used to estimate defect-resealing kinetics. GUVs (~0.8 mM lipid) were exposed to MAG2 (P/L = 1/300) for varying periods (0, 10, 60, or 120 min), and 20 μM external calcein was added to probe membrane permeability. Once a defect had resealed, calcein entry was blocked, resulting in a decreased fraction of calcein-positive GUVs. The samples were subsequently diluted with isotonic buffer (final calcein 1 μM) for flow cytometry analysis. The GUV population was gated using

FSC/SSC parameters as previously described.[1] Percentage of calcein-positive GUVs with different resealing time were quantified as:

Normalized % calcein-positive GUVs = $100 \times (P - P_0) / (P_{max} - P_0)$,

where P is the fraction of GUVs exhibiting calcein influx at each time point, P_0 is the percentage for calcein-positive GUVs incubated with calcein without MAG2, and P_{max} corresponds to percentage of calcein-positive GUVs exposed to MAG2 with no resealing time (0 min) after calcein addition.

Cell culture

KB cells were cultured on a 10 cm dish (JET BIOFIL) in Dulbecco's Modified Eagle Medium (DMEM) (Gibco) with 10% fetal bovine serum (FBS) (Biological Industries) and 1% Penicillin-Streptomycin (PS) (Biological Industries) at 5% CO_2 and 37 °C incubator for 2–3 days. To subculture KB cells, the cells were washed with PBS (pH 7.4) (Gibco) twice and added 1 ml of Trypsin-EDTA solution (Biological Industries) to incubate at 37 °C for 2 min. 2 ml of DMEM with 10% FBS was added, and the cell culture solution was then centrifuge at 200 × g for 5 min. The cell pellet was resuspended in a small volume of DMEM with 10% FBS and adopted 1/4 to 1/10 cells to a fresh 10 ml of DMEM with 10% FBS to subculture in a 10 cm dish.

Supplementary Table 1. AMP selected in this screening panel.

Peptides	Origin	Length	Net Charge	GRAVY Score	Bacterial Membrane-Lyticity	Hemolyticity
MAG2	African Clawed Frog	22	4.25	0.000	Moderate	Very Low
ScMAG2	Negative Control of MAG2	22	4.25	0.000	N/A (Artificially Designed in This Study)	
PEX	Magainin 2's Analog	26	8.00	0.019	High	Low/Moderate
DD1	Hylidae Frog	33	4.00	0.150	Moderate	Low/Moderate
EP1	Grouper	26	2.75	1.090	High	High
MEL	Honeybee	31	5.00	0.273	Extreme	High
MEL^m	Mutation of Melittin	29	5.00	0.588	N/A (Artificially Designed in This Study)	
TP4	Nile tilapia	30	7.75	-0.128	Extreme	High
TEML	European Red Frog	18	2.00	0.823	High	High

Net charge, bacterial membrane-lyticity, and hemolyticity of a peptide were cross-referenced from the Antimicrobial Peptide Database (APD3).[5] GRAVY score of a peptide was calculated using the ProtParam tool on the ExPASy server.[6]

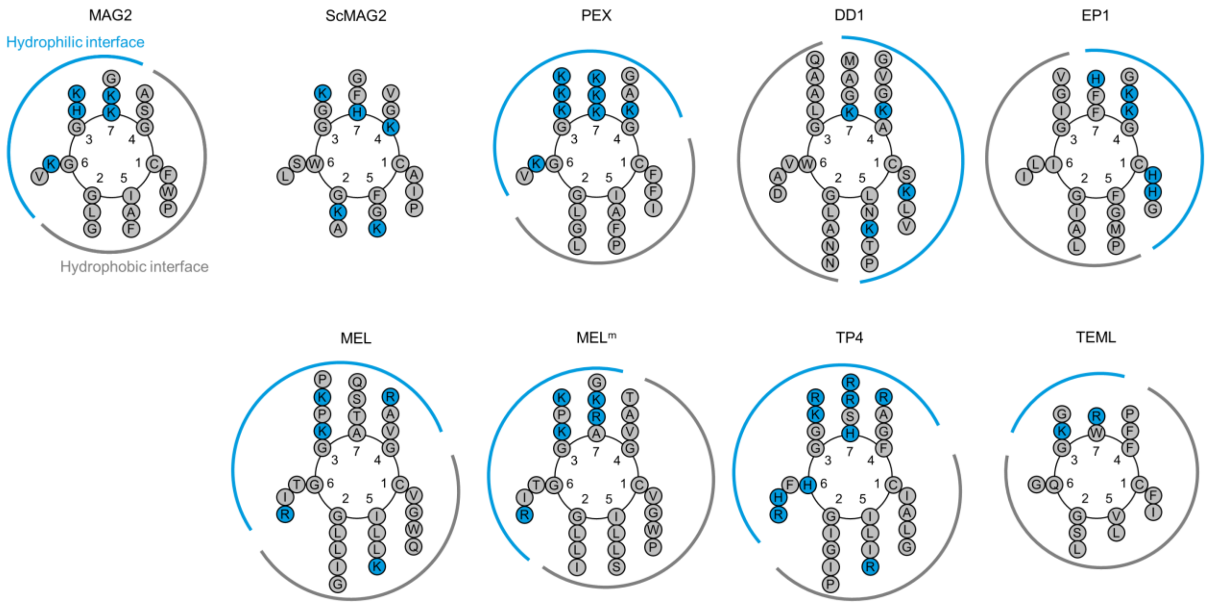

Supplementary Figure 1. Helical wheel diagrams of selected AMPs. Positively charged residues (Lys, Arg, and His) are shown in blue. Other residues are shown in gray.

a

Membrane lytic domain	Abbreviation	Sequence	Ionization	Annotate	Theoretical m/z	Observed m/z	Error (ppm)
Magainin 2	MAG2	CGGGIGKFLHSAKKWGKAFVGP-NH_2	MALDI	$[M + H]^+$	2244.2228	2244.2193	1.6
	4E-masked MAG2	CGGGIGKFLHSAKKWGKAFVGP-photolabile linker-EEEE-NH_2	ESI	$[M + 3H]^{3+}$	1014.1716	1014.1709	0.7
	8E-masked MAG2	CGGGIGKFLHSAKKWGKAFVGP-photolabile linker-EEEEEEEE-NH_2	ESI	$[M + 3H]^{3+}$	1186.2284	1186.2307	1.9
	12E-masked MAG2	CGGGIGKFLHSAKKWGKAFVGP-photolabile linker-EEEEEEEEEEEE-NH_2	ESI	$[M + 3H]^{3+}$	1358.2846	1358.2856	0.7

b

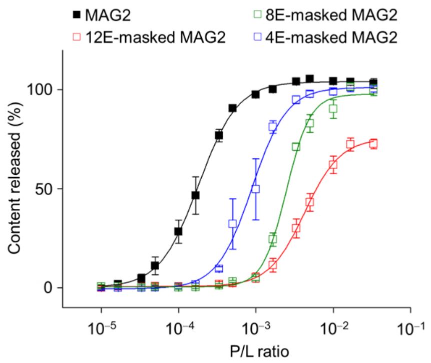

Supplementary Figure 2. (a) The mass of 4E, 8E, and 12E masked MAG2. (b) Covalent titration assay of MAG2 derivatives with different masking domain length. Different amounts of peptides were conjugated to the liposomes,

and the percentage of doxorubicin release was plotted against the P/L ratio. The $[P/L]_{50}$ values of MAG2, 12E-masked MAG2, 8E-masked MAG2, and 4E-masked MAG were 1.7×10^{-4}, 6.0×10^{-3}, 2.5×10^{-3}, and 8.8×10^{-4}, respectively. Data are presented as mean ± SD (n = 4). This result reveals that a twelve repeated glutamate provide satisfying masking in membrane-conjugated MAG2.

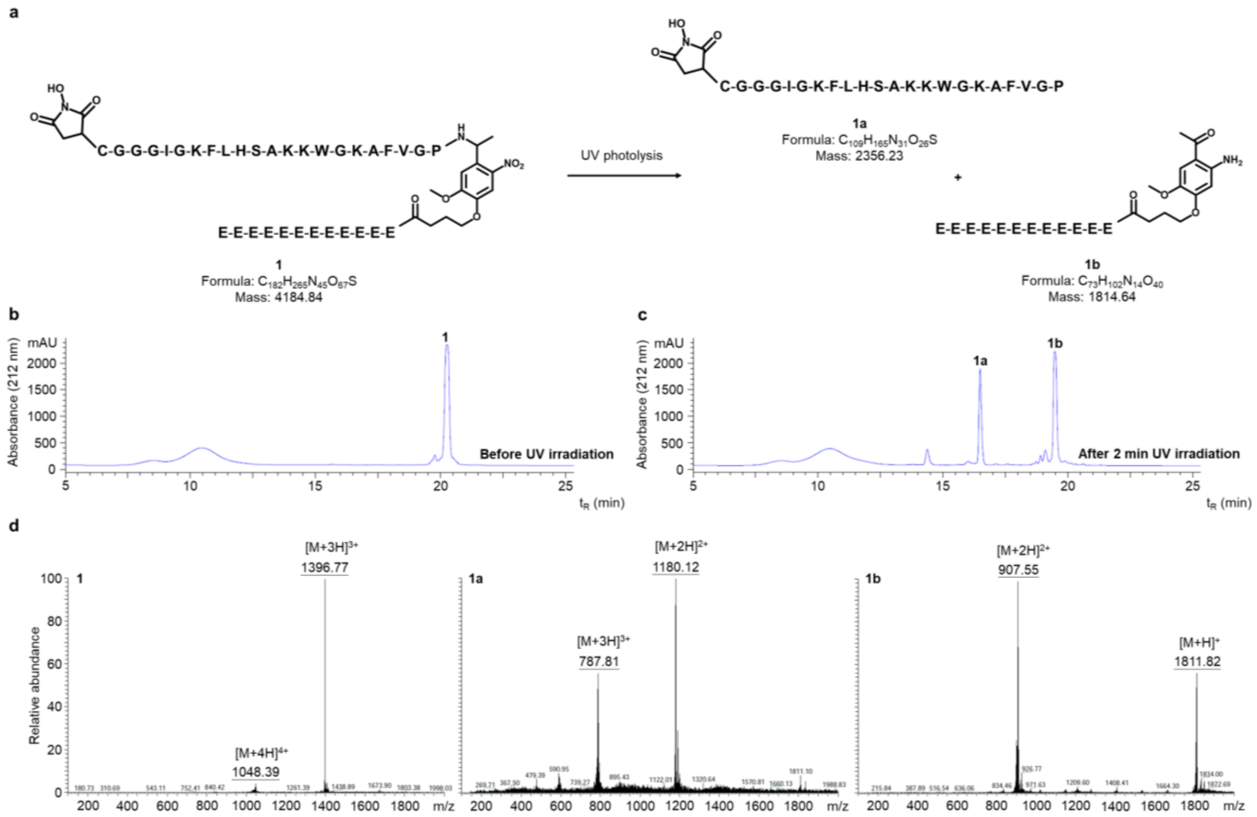

Supplementary Figure 3. (a) The photolysis reaction of the photolabile linker in 12E masked MAG2.[7] HPLC chromatograms of **1** (b) before irradiation or (c) after irradiation, using a linear elution gradient 10–90% solvent B over 40 min. The retention time (t_R) of **1** was 20.2 min, and the photolytic product fragments **1a** and **1b** appeared at t_R of 16.5 and 19.5 min, respectively. (d) ESI-MS spectra of **1**, **1a**, and **1b**.

Supplementary Table 2. The sequence and mass of unmasked and 12E masked antimicrobial peptides (AMPs).

Membrane lytic domain	Abbreviation	Sequence	Ionization	Annotate	Theoretical m/z	Observed m/z	Error (ppm)
Magainin 2	MAG2	CGGGIGKFLHSAKKWGKAFVGP-amide	MALDI	[M + H]$^+$	2244.2228	2244.2193	1.6
	4E masked MAG2	CGGGIGKFLHSAKKWGKAFVGP-photolabile linker-EEEE-amide	ESI	[M + 3H]$^{3+}$	1014.1716	1014.1709	0.7
	8E masked MAG2	CGGGIGKFLHSAKKWGKAFVGP-photolabile linker-EEEEEEEE-amide	ESI	[M + 3H]$^{3+}$	1186.2284	1186.2307	1.9
	Masked MAG2 (12E masked MAG2)	CGGGIGKFLHSAKKWGKAFVGP-photolabile linker-EEEEEEEEEEEE-amide	ESI	[M + 3H]$^{3+}$	1358.2846	1358.2856	0.7
Scrambled magainin 2	ScMAG2	CGGKFWHAKGGGSFIAKVKLGP	ESI	[M + 3H]$^{3+}$	749.0799	749.0764	4.7
	Masked scMAG2 (12E masked scMAG2)	CGGKFWHAKGGGSFIAKVKLGP-photolabile linker-EEEEEEEEEEEE-amide	ESI	[M + 4H]$^{4+}$	1018.9653	1018.9646	0.7
Pexiganan	PEX	CGGGIGKFLKKAKKFGKAFVKILKGP-amide	MALDI	[M + H]$^+$	2719.6689	2719.6610	2.9
	Masked PEX	CGGGIGKFLKKAKKFGKAFVKILKGP-photolabile linker-EEEEEEEEEEEE-amide	MALDI	[M + H]$^+$	4550.2911	4550.2745	3.6
DRS-DU-1	DD1	CGGALWKSLLKNVGKAAGKAALNAVTDMVNQGP-amide	MALDI	[M + H]$^+$	3281.7610	3281.7613	0.1
	Masked DD1	CGGALWKSLLKNVGKAAGKAALNAVTDMVNQGP-photolabile linker-EEEEEEEEEEEE-amide	ESI	[M + 4H]$^{4+}$	1278.3505	1278.3481	1.9
Epinecidin-1	EP1	CGGGFIFHIIKGLFHAGKMIHGLVGP-amide	MALDI	[M + H]$^+$	2705.4689	2705.4666	0.8
	Masked EP1	CGGGFIFHIIKGLFHAGKMIHGLVGP-photolabile linker-EEEEEEEEEEEE-amide	ESI	[M + 3H]$^{3+}$	1512.0333	1512.0356	1.5
Melittin	MEL	CGGGIGAVLKVLTTGLPALISWIKRKRQQGP-amide	MALDI	[M + H]$^+$	3216.8883	3216.8840	1.3
	Masked MEL	CGGGIGAVLKVLTTGLPALISWIKRKRQQGP-photolabile linker-EEEEEEEEEEEE-amide	ESI	[M + 4H]$^{4+}$	1262.1317	1262.1319	0.2
Melittin-mutation	MEL^m	CGGGIGAVLKVLTRGLPALIKWIKTSRGP-amide	ESI	[M + 3H]$^{3+}$	987.5951	987.5967	1.6
	Masked MEL^m	CGGGIGAVLKVLTRGLPALIKWIKTSRGP-photolabile linker-EEEEEEEEEEEE-amide	ESI	[M + 4H]$^{4+}$	1198.1024	1198.1049	2.1
Tilapia Piscidin 4	TP4	CGGFIHHIIGGLFSAGKAIHRLIRRRRRGP-amide	MALDI	[M + H]$^+$	3350.9350	3350.9331	0.6
	Masked TP4	CGGFIHHIIGGLFSAGKAIHRLIRRRRRGP-photolabile linker-EEEEEEEEEEEE-amide	ESI	[M + 4H]$^{4+}$	1295.6433	1295.6450	1.3
Temporin L	TEML	CGGFVQWFSKFLGRILGP-amide	MALDI	[M + H]$^+$	2011.0740	2011.0732	0.4
	Masked TEML	CGGFVQWFSKFLGRILGP-photolabile linker-EEEEEEEEEEEE-amide	MALDI	[M + H]$^+$	3839.6910	3839.6875	0.9

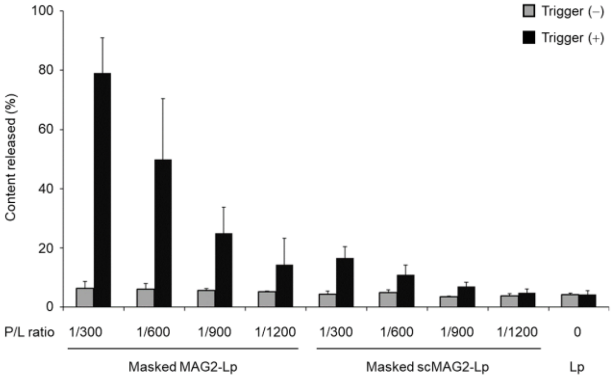

Liposome	P/L ratio	Content release without trigger (%)	Content release with trigger (%)
Masked MAG2-Lp	1/300	6 ± 2	79 ± 11
	1/600	6 ± 2	50 ± 20
	1/900	6 ± 1	25 ± 9
	1/1200	5 ± 0	14 ± 9
Masked scMAG2-Lp	1/300	4 ± 1	17 ± 4
	1/600	5 ± 1	11 ± 3
	1/900	4 ± 0	7 ± 1
	1/1200	4 ± 1	5 ± 1
Lp	-	4 ± 0	4 ± 1

Supplementary Figure 4. Trigger-release profiles of masked MAG2 liposomes with different P/L ratios ranging from 1/1200 to 1/300, followed by incubation at 37 °C for 1 h. The detail release data are provided in the adjacent table as mean ± SD (n = 3). **Masked MAG2-Lp** at a P/L ratio of 1/300 displayed a significant content release (79%) after trigger. In contrast, **masked scMAG2-Lp** at a P/L ratio of 1/300 showed 17% after trigger. The negative control **Lp** demonstrated no release upon trigger, confirming that trigger responsiveness requires masked MAG2 conjugation. These findings identify a P/L ratio of 1/300 is suitable for generating **masked MAG2-Lp**.

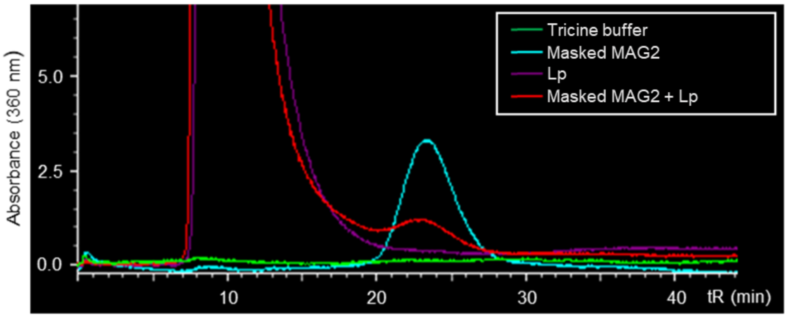

Supplementary Figure 5. Determination of peptide conjugation efficiency via size exclusion chromatography. The conjugation efficiency of **masked MAG2Lp** (P/L ratio = 1/300) was analyzed using a CL-4B column on an HPLC system. Based on the integrated peak area of the peptide fraction (t_R = 23.5 min), the conjugation efficiency was determined to be approximately 80%.

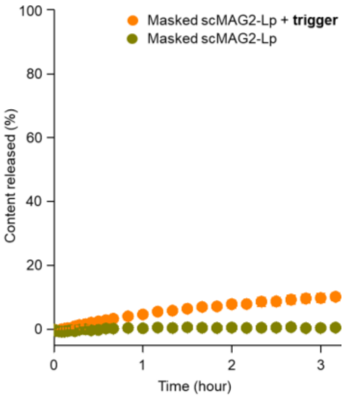

Supplementary Figure 6. Trigger-release profiles of **masked scMAG2-Lp** with or without trigger at 37 °C. Data are presented as mean ± SD (n = 3).

Supplementary Table 3. DLS measurements of liposome particle size, zeta potential, and particle concentration.

Samples	Diameter (nm)	PDI	Zeta potential (mV)	Particle concentration (number/ml)
Lp (doxorubicin free)	132 ± 36	0.05 ± 0.01	-2.2 ± 0.6	
Lp	132 ± 39	0.07 ± 0.03	-2.3 ± 0.2	
Lp + **trigger**	132 ± 42	0.09 ± 0.02	-2.5 ± 0.2	
Masked MAG2-Lp	131 ± 34	0.04 ± 0.02	-6.7 ± 0.8	4.96×10^9
Masked MAG2-Lp + **trigger**	131 ± 36	0.06 ± 0.04	-2.9 ± 0.8	5.28×10^9
Masked scMAG2-Lp	133 ± 33	0.04 ± 0.00	-6.2 ± 0.2	
Masked scMAG2-Lp + **trigger**	132 ± 41	0.09 ± 0.01	-1.7 ± 0.4	

Supplementary Table 4. DLS measurements of liposome particle size, zeta potential, and particle concentration of **masked MAG2-Lp** with P/L ratios from 1/600 to 1/1200.

Samples	Diameter (nm)	PDI	Zeta potential (mV)
Masked MAG2-Lp (P/L ratio = 1/600)	133 ± 36	0.06 ± 0.02	-4.2 ± 0.9
Masked MAG2-Lp (P/L ratio = 1/600) + **trigger**	134 ± 39	0.05 ± 0.02	-3.3 ± 0.2
Masked MAG2-Lp (P/L ratio = 1/900)	132 ± 42	0.05 ± 0.01	-5.0 ± 2.0
Masked MAG2-Lp (P/L ratio = 1/900) + **trigger**	133 ± 34	0.07 ± 0.01	-2.9 ± 0.5
Masked MAG2-Lp (P/L ratio = 1/1200)	133 ± 36	0.05 ± 0.02	-3.2 ± 0.5
Masked MAG2-Lp (P/L ratio = 1/1200) + **trigger**	133 ± 33	0.05 ± 0.02	-3.2 ± 0.6

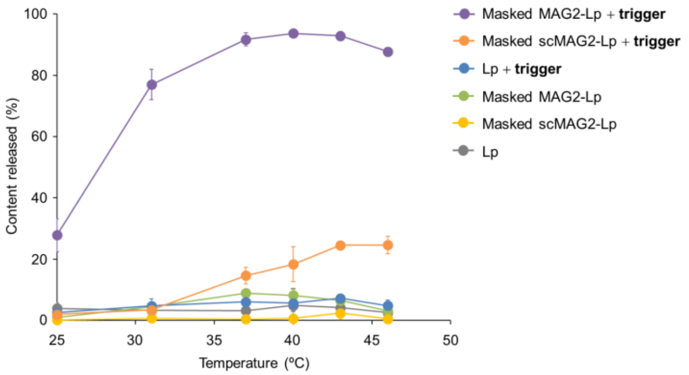

Supplementary Figure 7. Temperature-dependent trigger-release profiles of **masked MAG2-Lp** with or without trigger, followed by incubation at different temperatures (25−46 °C) for 1 h. Data are presented as mean ± SD (n = 2).

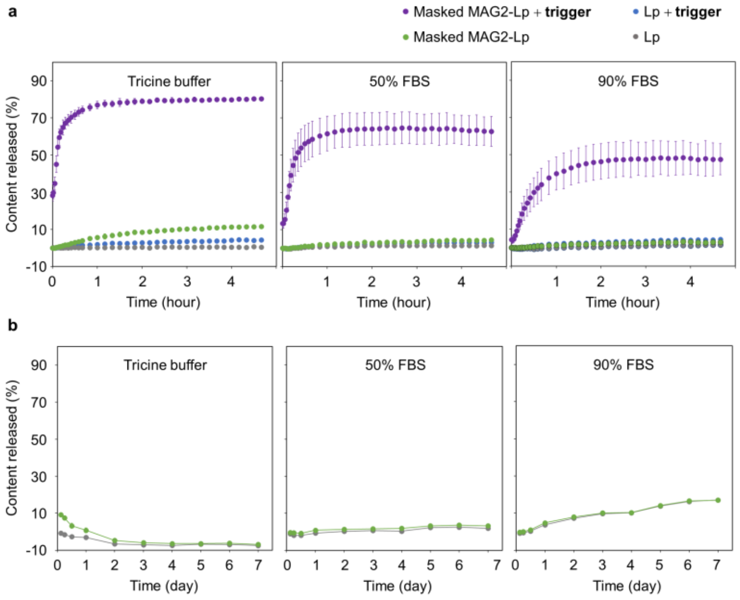

Supplementary Figure 8. (a) Serum concentration-dependent trigger-release profiles of **masked MAG2-Lp**, followed by incubation at 37 °C for 5 h. Data are presented as mean ± SD (n = 3). (b) Serum concentration-dependent long term encapsulation stability of **masked MAG2-Lp** at 37 °C. Data are presented as mean ± SD (n = 3).

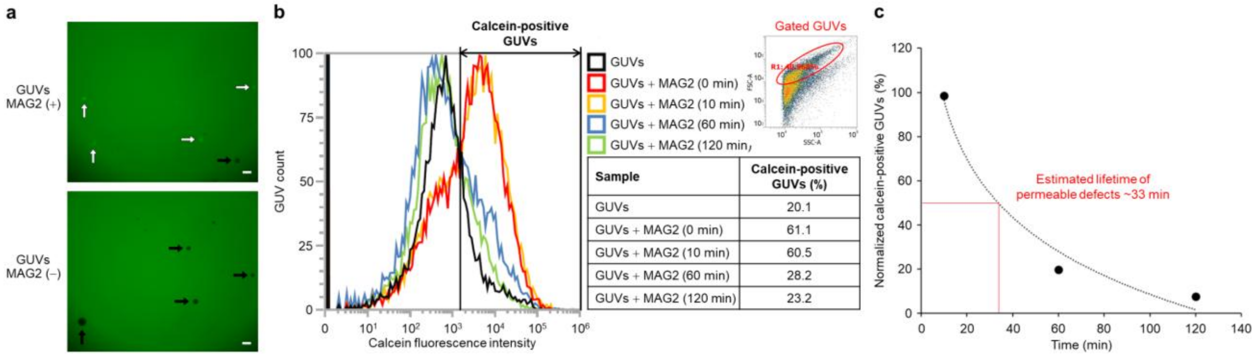

Supplementary Figure 9. (a) Fluorescence microscopy visualization of GUV calcein influx by membrane defect (white arrow) and calcein influx blockage by defect resealing (black arrow). Large GUVs (> 8 μm) were visually selected for analysis. GUVs incubate with MAG2 for 1h have membrane disruption and resealing, showing 29 calcein-positive GUVs over 43 of total GUVs (67%), and GUVs without MAG2 incubation showing 3 calcein-positive GUVs over 50 of total GUVs (6%). Scale bar: 20 μm. (b) Flow cytometry analysis of calcein-positive GUVs population (region within black double arrow). Inset: The dashed red box represents the FSC/SSC-gated GUVs for analysis. The percentages of calcein-positive GUVs are shown in the adjacent table. (c) Fitting curve of normalized percentage of calcein-positive GUVs over allowed membrane resealing time. The estimated lifetime of permeable defects is ~33 min.

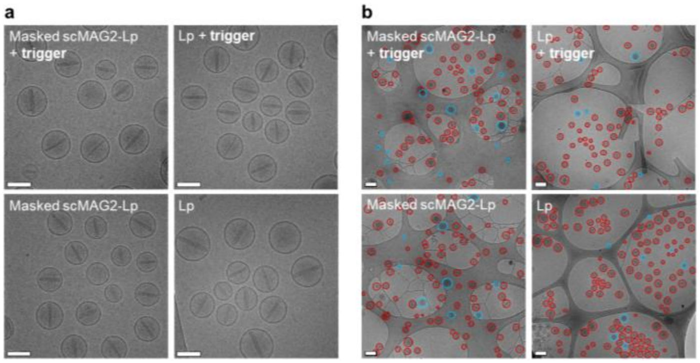

Supplementary Figure 10. (a) Cryo-EM micrographs of **masked scMAG2-Lp** and **Lp** with or without trigger at 50000× magnification. Scale bar: 100 nm. (b) Content release estimation (red: doxorubicin remained liposomes. Blue: empty liposomes) of **masked scMAG2-Lp** and **Lp** with or without trigger at 5000× magnification. Empty liposome percentage of **masked scMAG2-Lp** with trigger, **masked scMAG2-Lp** without trigger, **Lp** with trigger, and **Lp** without trigger are 17% (27/158), 9% (18/198), 8% (14/176), and 6% (8/130), respectively. Scale bar: 200 nm.

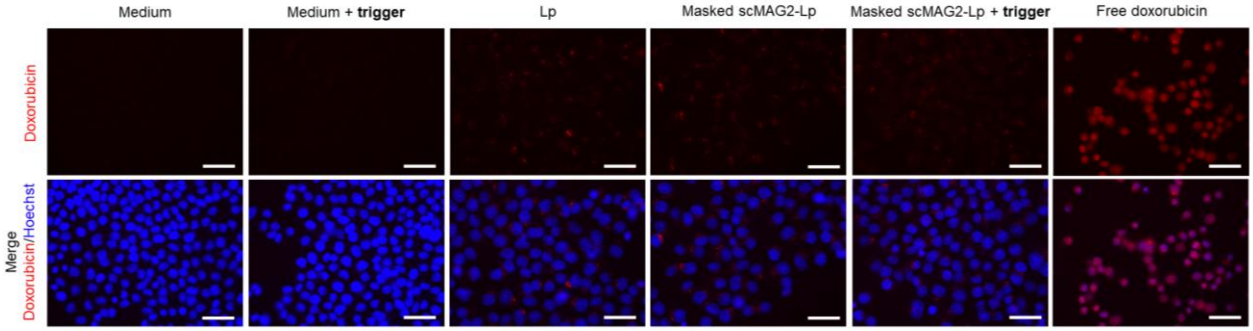

Supplementary Figure 11. Fluorescence images of KB cells treated with negative control **Lp** and **masked scMAG2-Lp** with or without trigger at 37°C for 20 h. Merged images show doxorubicin fluorescence (red) and nucleus Hoechst staining (blue). Scale bar: 100 µm.

Supplementary Table 5. Statistical analysis of the positive peptide lateral aggregates on liposomes based on cryo-EM images.

**Sample name**	**Empty liposomes / Total liposomes**	**Peptide aggregated liposomes / Total liposomes**	**Peptide aggregated liposomes / Empty liposomes**
Lp	2/160	0/160	0/2
Lp + **trigger**	4/166	0/166	0/4
Masked MAG2-Lp	15/182	6/182	0/15
Masked MAG2-Lp + **trigger**	151/183	62/183	54/151
Masked scMAG2-Lp	12/169	1/169	0/12
Masked scMAG2-Lp + **trigger**	12/166	18/166	1/12

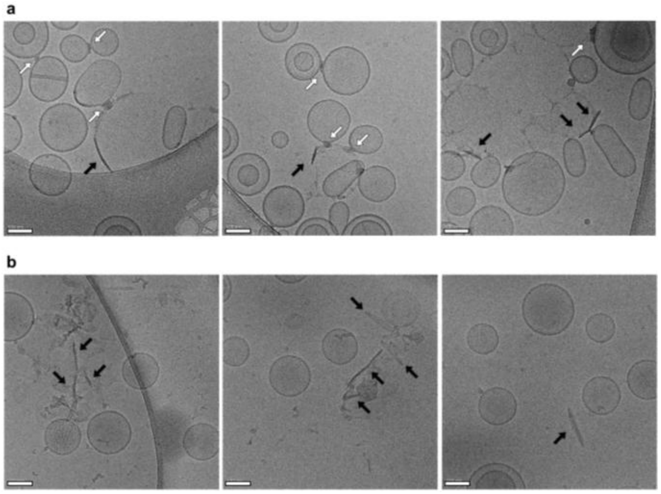

Supplementary Figure 12. Liposome fragmentation by high peptide conjugation concentration (P/L ratio = 1/100) visualized by (a) representative positive staining cryo-EM micrographs of **masked MAG2-Lp** with trigger (triplicate) and (b) representative cryo-EM micrographs of **masked MAG2-Lp** with trigger (triplicate). White arrows point to UA-stainable peptide clusters on the liposome surface, while black arrows point to membrane fragments. Scale bar: 100 nm.

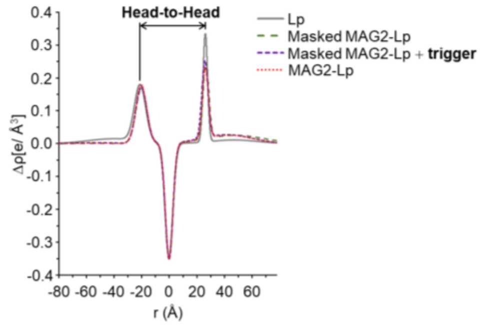

Supplementary Figure 13. The full-scale electron density (ED) profiles fitted from the SAXS data, where the Head-to-Head (black double arrow) distance plus the hydration layer represents the membrane thickness.

References

1. Fujii, S.; Matsuura, T.; Sunami, T.; Nishikawa, T.; Kazuta, Y.; Yomo, T., Liposome display for in vitro selection and evolution of membrane proteins. *Nat Protoc* **2014,** *9* (7), 1578-91.
2. Moga, A.; Yandrapalli, N.; Dimova, R.; Robinson, T., Optimization of the Inverted Emulsion Method for High-Yield Production of Biomimetic Giant Unilamellar Vesicles. *Chembiochem* **2019,** *20* (20), 2674-2682.
3. Tamba, Y.; Yamazaki, M., Single giant unilamellar vesicle method reveals effect of antimicrobial peptide magainin 2 on membrane permeability. *Biochemistry-Us* **2005,** *44* (48), 15823-15833.

4. Dey, S.; Dorey, A.; Abraham, L.; Xing, Y. Z.; Zhang, I.; Zhang, F.; Howorka, S.; Yan, H., A reversibly gated protein-transporting membrane channel made of DNA. *Nat Commun* **2022,** *13* (1).

5. Wang, G. S.; Li, X.; Wang, Z., APD3: the antimicrobial peptide database as a tool for research and education. *Nucleic Acids Res* **2016,** *44* (D1), D1087-D1093.

6. Wilkins, M. R.; Gasteiger, E.; Bairoch, A.; Sanchez, J. C.; Williams, K. L.; Appel, R. D.; Hochstrasser, D. F., Protein identification and analysis tools in the ExPASy server. *Methods Mol Biol* **1999,** *112*, 531-52.

7. O'Hagan, M. P.; Duan, Z. J.; Huang, F. J.; Laps, S.; Dong, J. T.; Xia, F.; Willner, I., Photocleavable-Nitrobenzyl-Protected DNA Architectures and Their Applications. *Chem Rev* **2023,** *123* (10), 6839-6887.
